# CEH-60/PBX regulates vitellogenesis and cuticle permeability through intestinal interaction with UNC-62/MEIS in *Caenorhabditis elegans*

**DOI:** 10.1371/journal.pbio.3000499

**Published:** 2019-11-01

**Authors:** Pieter Van de Walle, Ellen Geens, Geert Baggerman, Francisco José Naranjo-Galindo, Peter Askjaer, Liliane Schoofs, Liesbet Temmerman

**Affiliations:** 1 Animal Physiology and Neurobiology, University of Leuven (KU Leuven), Leuven, Belgium; 2 Centre for Proteomics (CFP), University of Antwerp, Antwerpen, Belgium; 3 VITO, Mol, Belgium; 4 Andalusian Center for Developmental Biology (CABD), Universidad Pablo de Olavide, Seville, Spain; University of Massachusetts Medical School, UNITED STATES

## Abstract

The onset of sexual maturity involves dramatic changes in physiology and gene expression in many animals. These include abundant yolk protein production in egg-laying species, an energetically costly process under extensive transcriptional control. Here, we used the model organism *Caenorhabditis elegans* to provide evidence for the spatiotemporally defined interaction of two evolutionarily conserved transcription factors, CEH-60/PBX and UNC-62/MEIS, acting as a gateway to yolk protein production. Via proteomics, bimolecular fluorescence complementation (BiFC), and biochemical and functional readouts, we show that this interaction occurs in the intestine of animals at the onset of sexual maturity and suffices to support the reproductive program. Our electron micrographs and functional assays provide evidence that intestinal PBX/MEIS cooperation drives another process that depends on lipid mobilization: the formation of an impermeable epicuticle. Without this lipid-rich protective layer, mutant animals are hypersensitive to exogenous oxidative stress and are poor partners for mating. Dedicated communication between the hypodermis and intestine in *C*. *elegans* likely supports these physiological outcomes, and we propose a fundamental role for the conserved PBX/MEIS interaction in multicellular signaling networks that rely on lipid homeostasis.

## Abstract 2

The evolutionarily conserved interaction of two Hox cofactors (PBX and MEIS orthologues) in the nematode Caenorhabditis elegans controls the production of yolk and renders the animal's surface coat impermeable to small molecules.

## Introduction

Vitellogenesis is the process of maternal yolk formation. In many organisms, including insect and vertebrate representatives [1,2], it is deemed essential for embryonic development, although the necessity of abundant yolk synthesis for development has been questioned in some species, including *C*. *elegans* [3,4]. Vitellogenins (VITs) are precursors to yolk proteins and are synthesized in the *C*. *elegans* intestine. After they have been released in the body cavity, yolk proteins can be taken up by the oocytes by receptor-mediated endocytosis [5].

Given the significant cost of yolk protein production for a growing organism, the onset of vitellogenesis is under tight transcriptional control [6]. For example, insulin signaling and dietary restriction in *C*. *elegans* are known to respectively affect *vit* transcription and yolk provisioning to embryos [7–9], indicating that environmental cues play a role in regulating yolk protein production. Several intestinal transcription factors, including UNC-62 (*unc*oordinated), ELT-2 (*e*rythroid-*l*ike *t*ranscription factor), and MAB-3 (*m*ale *ab*normal), bind directly to the promoter region of *vit* genes [9,10]. In earlier work, we recovered mutants of a transcription factor, CEH-60 (*C. elegans* *h*omeobox), that produced almost no yolk proteins. It is remarkable that several mutants for regulators of vitellogenesis, including CEH-60, do not show any defects in fertility, development, or viability [3,11,12]. So far, no cell non-autonomous mechanism could be suggested that would explain CEH-60’s control of vitellogenesis [3].

CEH-60 is a member of the pre–B cell leukemia (PBC)-class of Hox cofactors, which also comprises CEH-20 and CEH-40 [13]. The latter two have been implicated in hypodermal and neuronal development, for which they interact with the myeloid ecotropic viral integration site (MEIS)-class transcription factor UNC-62 [14,15]. Existing data may support proposing UNC-62 as a potential partner for CEH-60 in the case of vitellogenesis as well: lower levels of *vit* gene transcripts have been observed upon *unc-62* RNA interference (RNAi) treatment, and UNC-62 binds directly to the promoters of *vit* genes [16]. UNC-62 is a known target of a developmental microRNA pathway that is switched on during sexual adulthood. It signals from the zinc transcription factor LIN-29 (abnormal cell *lin*eage) in the hypodermis through TORC2 (*t*arget *o*f *r*apamycin *c*omplex 2), SGK-1 (*s*erum- and *g*lucocorticoid- inducible *k*inase homolog), and PQM-1 (*p*araquat (*m*ethylviologen) *r*esponsive) in the intestine to ultimately affect timely transcriptional activation of *vit* genes through UNC-62, indicating an interplay between the hypodermis and intestine in the regulation of vitellogenesis [11]. However, while UNC-62 is broadly expressed in neurons, intestine, seam cells, and hypodermis [17,18], expression of *ceh-60* seemed limited to a single pair of sensory neurons [3,19]. This raises doubt about the possibility of direct interaction and poses important questions as to how CEH-60 might regulate intestinal vitellogenesis.

Here, we show that the earlier-reported expression of *ceh-60* in one pair of head neurons is incomplete. We identify the neuron pair as amphid wing “C” (AWC), in which expression is observed throughout life, and additionally reveal expression in pharyngeal muscle cells (pm6) and intestine. Notably, abundant *ceh-60* expression in the intestine is only observed from the L4 larval stage onwards. In line with this observation, we provide evidence for direct in vivo interaction between UNC-62 and CEH-60 in the adult intestine. This interaction depends on CEH-60’s PBC domain and is responsible for abundant VIT production. As a PBC-class Hox cofactor, we hypothesized that CEH-60 would also be involved in other processes. Based on differential proteomics data, we can propose roles in lipid metabolism, immune function, and stress resistance. Most strikingly, combined with loss-of-function data, CEH-60 proves pivotal for proper development of an impermeable cuticle. Because this phenotype also depends on intestinal interaction of CEH-60 and UNC-62, our work provides evidence for a framework in which CEH-60/PBX (pre B-cell leukemia homeobox) and UNC-62/MEIS co-operate in the intestine to control different physiological outcomes.

## Results

### *ceh-60* mutants skimp on VITs, lipid metabolic proteins, and cuticle collagens

To gain an unbiased insight into global molecular changes occurring in the yolk-deprived *ceh-60* mutants, we performed a differential proteomics experiment comparing wild-type animals with animals carrying the *ceh-60(lst466)* nonsense mutation. We identified 1,749 proteins, 17 of which were differentially down- and 14 differentially up-regulated (fold changes <0.75 or >1.25, and *p* < 0.05, Table 1). As expected [3], all 6 VIT proteins were drastically down-regulated, and they emerged as the 6 most differentially regulated proteins in our dataset. Excluding VITs, the most strongly down-regulated proteins are cuticle collagens (COL) that are part of the impermeable extracellular matrix: COL-120 (fold change 0.56) and COL-106 (fold change 0.72) (Table 1). Other down-regulated proteins are involved in lipid metabolism, including isovaleryl-CoA dehydrogenase (IVD-1) [20] and the short-chain fatty acid breakdown enzymes hydroxyacyl-CoA hydrolase (HACH-1) and acyl-CoA dehydrogenase (ACDH-1) [21], suggesting an altered lipid metabolism as a consequence of disrupted expression of lipid-binding yolk proteins. Indeed, we observed an increased accumulation of intestinal fat in adult *ceh-60* mutants by Oil-Red-O staining, which becomes more pronounced with aging (S1 Fig).

**Table 1:** Proteins up- or down-regulated in *ceh-60* mutants versus wild type.

Down-regulated (mutant versus wild type)					
**Protein ID**	**Protein**	**Gene name**	**Fold change**	***p*-value**	**Fold change RNAseq [12]**
VIT5_CAEEL	Vitellogenin 5	*vit-5*	0.15	<0.0001	0.035
VIT4_CAEEL	Vitellogenin 4	*vit-4*	0.16	<0.0001	0.012
VIT3_CAEEL	Vitellogenin 3	*vit-3*	0.16	<0.0001	0.048
VIT2_CAEEL	Vitellogenin 2	*vit-2*	0.20	<0.0001	0.161
VIT6_CAEEL	Vitellogenin 6	*vit-6*	0.22	<0.0001	0.053
VIT1_CAEEL	Vitellogenin 1	*vit-1*	0.27	<0.0001	0.109
Q19813_CAEEL	Collagen-120	*col-120*	0.56	<0.0001	-
CALM_CAEEL	Calmodulin	*cmd-1*	0.56	<0.0001	-
G5EEH6_CAEEL	Isovaleryl-CoA dehydrogenase	*ivd-1*	0.66	0.0008	-
PSMD3_CAEEL	26S proteasome non-ATPase regulatory subunit 3	*rpn-3*	0.68	0.0023	-
Q8MNT7_CAEEL	Hydroxyacyl-coA hydrolase	*hach-1*	0.69	0.005	-
ULE4_CAEEL	UPF0375 protein	*ule-4*	0.72	0.0156	0.760
Q8MXT6_CAEEL	Collagen-106	*col-106*	0.73	0.0201	1.433
O17685_CAEEL	Uncharacterized protein	*C49F5*.*7*	0.73	0.0201	-
O44145_CAEEL	Permeable eggshell	*perm-2*	0.74	0.0277	-
C2BR91_CAEEL	Cystatin	*C39B5*.*5*	0.74	0.028	2.443
H2KZG6_CAEEL	Acyl-CoA dehydrogenase	*acdh-1*	0.75	0.028	-
Up-regulated (mutant versus wild type)					
**Protein ID**	**Protein**	**Gene name**	**Fold change**	***p*-value**	**Fold change RNAseq** [12]
PGP1_CAEEL	Multidrug resistance protein 1	*pgp-1*	1.94	<0.0001	3.870
O45444_CAEEL	C-type lectin 63	*clec-63*	1.54	0.0005	3.522
O18307_CAEEL	Uncharacterized protein	*ZK909*.*3*	1.48	0.0021	-
RB11A_CAEEL	Ras-related protein Rab11.1	*rab-11*.*1*	1.44	0.0052	-
O76367_CAEEL	Cytochrome Oxidase assembly protein	*cox-6c*	1.38	0.0201	1.214
Q17475_CAEEL	Uncharacterized protein	*B0334*.*3*	1.38	0.0197	1.473
O17621_CAEEL	Uncharacterized protein	*C29F7*.*2*	1.35	0.0277	2.849
L8E833_CAEEL	Uncharacterized protein	*W05H9*.*1*	1.34	0.028	1.911
Q18577_CAEEL	Uncharacterized protein	*C42D4*.*1*	1.32	0.0367	1.402
GST7_CAEEL	Probable glutathione S-transferase 7	*gst-7*	1.3	0.0395	1.168
Q22562_CAEEL	Uncharacterized protein	*T19B10*.*2*	1.3	0.0395	1.479
P4HA1_CAEEL	Prolyl 4-hydroxylase subunit alpha-1	*dpy-18*	1.29	0.0395	-
IFB2_CAEEL	Intermediate filament protein B2	*ifb-2*	1.27	0.0395	1.900
PDIA4_CAEEL	Probable protein disulfide-isomerase A4	*C14B9*.*2*	1.26	0.0395	1.915

We identified respectively 17 and 14 differentially down- and up-regulated proteins (fold changes <0.75 or >1.25) in *ceh-60(lst466)*. The 6 VITs are the most down-regulated. Cuticle collagens (COL-120 and COL-106) and fatty acid metabolism proteins (IVD-1, HACH-1, and ACDH-1) represent other down-regulated proteins. Two known immune defense-related proteins (PGP-1, CLEC-63) are up-regulated. RNAseq fold changes from *ceh-60(ok1485)* versus control [12] are shown for comparison. Transcripts for which no significant change was reported in the RNAseq dataset (*p* < 0.01) are marked with “-.”

Abbreviation: CoA, coenzyme A.

Some stress and immune defense proteins are more abundant in *ceh-60(lst466)* mutants than in controls. These include PGP-1 (*P g*lycoprotein *r*elated), an ATP-binding membrane transporter, CLEC-63 (*C*-type *l*ectin), and GST-7 (*g*lutathione *S*-*t*ransferase), all of which play important roles in bacterial pathogen response [22–25]. Taken together, this confirms the ability of CEH-60 to regulate VIT levels and suggests new roles for CEH-60 in cuticle structure and stress response.

We compared our proteomics results with recent RNA sequencing (RNAseq) data of the *ceh-60(ok1485)* allele [12] and found that 18 out of 20 proteins with a deregulated transcript in the RNAseq dataset show regulation in the same direction (Table 1; *p* = 0.01). This indicates that the vast majority of proteome changes occurring after CEH-60 disruption result directly from changes at the transcriptional level. Clearly, mutants carrying either *ceh-60* allele respond similarly to loss of functional CEH-60.

### *ceh-60* is expressed in AWC neurons, intestine, and pharyngeal muscle

**Fig 1:**
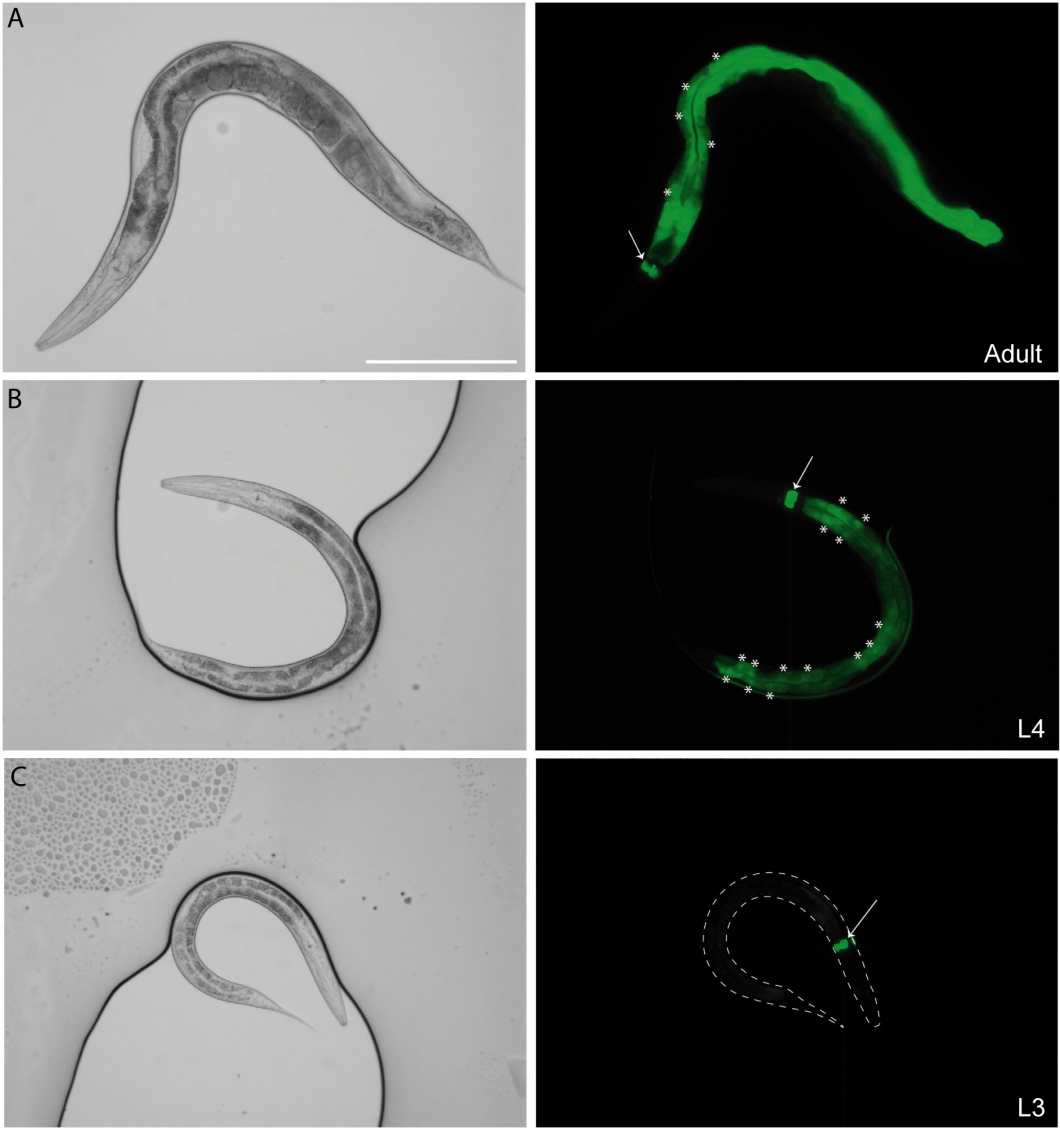
*ceh-60* is expressed in the intestine from the L4 larval stage onwards. Bright-field and GFP images of a *ceh-60p*::*ceh-60*::*SL2*::*gfp* reporter strain showing expression in the intestine (*) and pharynx (arrow) of (A) adult and (B) L4 animals. Intestinal expression is weaker in L4 larvae than in adults. (C) Expression in the intestine is not clearly observed in the L3 larval stage or before. Neuronal expression, while present (S2 Fig), is not clearly visible at this magnification. White dotted lines mark the outline of the L3 animal. Scale bar, 200 μm. GFP, green fluorescent protein.

**Fig 2:**
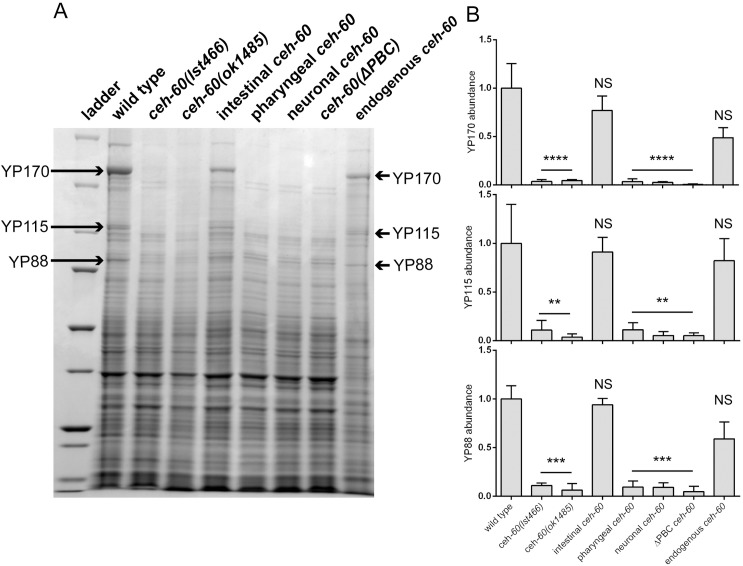
Intestinal expression and an intact PBC domain are essential for CEH-60’s vitellogenesis-regulating function. (A) Upon electrophoresis of total protein extracts, yolk proteins (YP170, YP115, and YP88) are present as abundant bands in wild-type animals but not in *ceh-60(lst466)* or in *ceh-60(ok1485)*. Restoring *ceh-60* expression under its own promoter (*ceh-60p*::*ceh-60*) or in the intestine (*elt-2p*::*ceh-60*) rescues the presence of yolk proteins in *ceh-60(lst466)* mutants, but expressing *ceh-60* in the AWC neurons (*odr-1p*::*ceh-60*) or the pharyngeal muscles (*myo-2p*::*ceh-60*) does not. Truncating the PBC domain of resupplied CEH-60 (*ceh-60p*::*ceh-60(ΔPBC)*) also does not rescue yolk protein production in *ceh-60* mutants. Yolk protein band identity is based on [7,27]. (B) Quantification of yolk proteins YP170, YP115, and YP88, normalized against total protein present in a lane and rescaled so that each YP has a mean abundance of 1 in wild type. **p < 0.01, ***p < 0.001, ****p < 0.0001. Error bars = SEM. *N* ≥ 3. Underlying data are available in S1 Data. NS, not significant; YP, yolk protein.

An existing transcriptional construct of the *ceh-60* promoter fused to green fluorescent protein (*gfp*) shows expression in a single pair of amphid neurons [19]. Through crossing *ceh-60p*::*ceh-60*::*gfp* animals with a marker strain carrying the *odr-1p*::*rfp* transgene, we identified this pair as the olfactory AWC neurons (S2A Fig). While it is certainly possible that effects of CEH-60 on intestinal VIT levels may be indirect, the limited expression pattern of this transcription factor demands confirmation in light of the above. Therefore, we constructed a strain carrying a longer 3.5-kb *ceh-60* promoter sequence followed by the *ceh-60* coding region, an SL2 *trans*-splicing sequence and *gfp*. We also relied on a fosmid carrying the full genomic *ceh-60* locus followed by *gfp* and flanked by large regions of endogenous 5′ (3.5-kb) and 3′ (4.1-kb) neighboring sequences [26]. Both constructs confirmed expression in the AWC neurons but also included the pm6 pharyngeal muscle cells (identity based on their typical three-lobed morphology) and intestinal nuclei (S2B and S2C Fig). Clear intestinal reporter expression was only observed as of the L4 larval stage (Fig 1), indicating temporal control and coinciding with the onset of vitellogenesis. In search for the tissue in which CEH-60 acts to regulate vitellogenesis, we used promoters of genes that are strongly expressed in the intestine (*elt-2p*), in pharyngeal muscle (*myo-2p*), or in AWC neurons (*odr-1p*) to drive *ceh-60* expression in *ceh-60(lst466)* mutants. In line with expectations, intestinal expression (*elt-2p*::*ceh-60*) suffices to rescue the vitellogenesis-deficient phenotype of *ceh-60(lst466)* animals (Fig 2), while expressing *ceh-60* in the AWC neurons (*odr-1p*::*ceh-60*) or pharyngeal muscle (*myo-2p*::*ceh-60*) fails to do so (Fig 2). This indicates that the site of action of CEH-60 as a regulator of vitellogenesis is the intestine.

### Interaction of CEH-60 with UNC-62 in the adult intestine promotes *vit* gene expression

**Fig 3:**
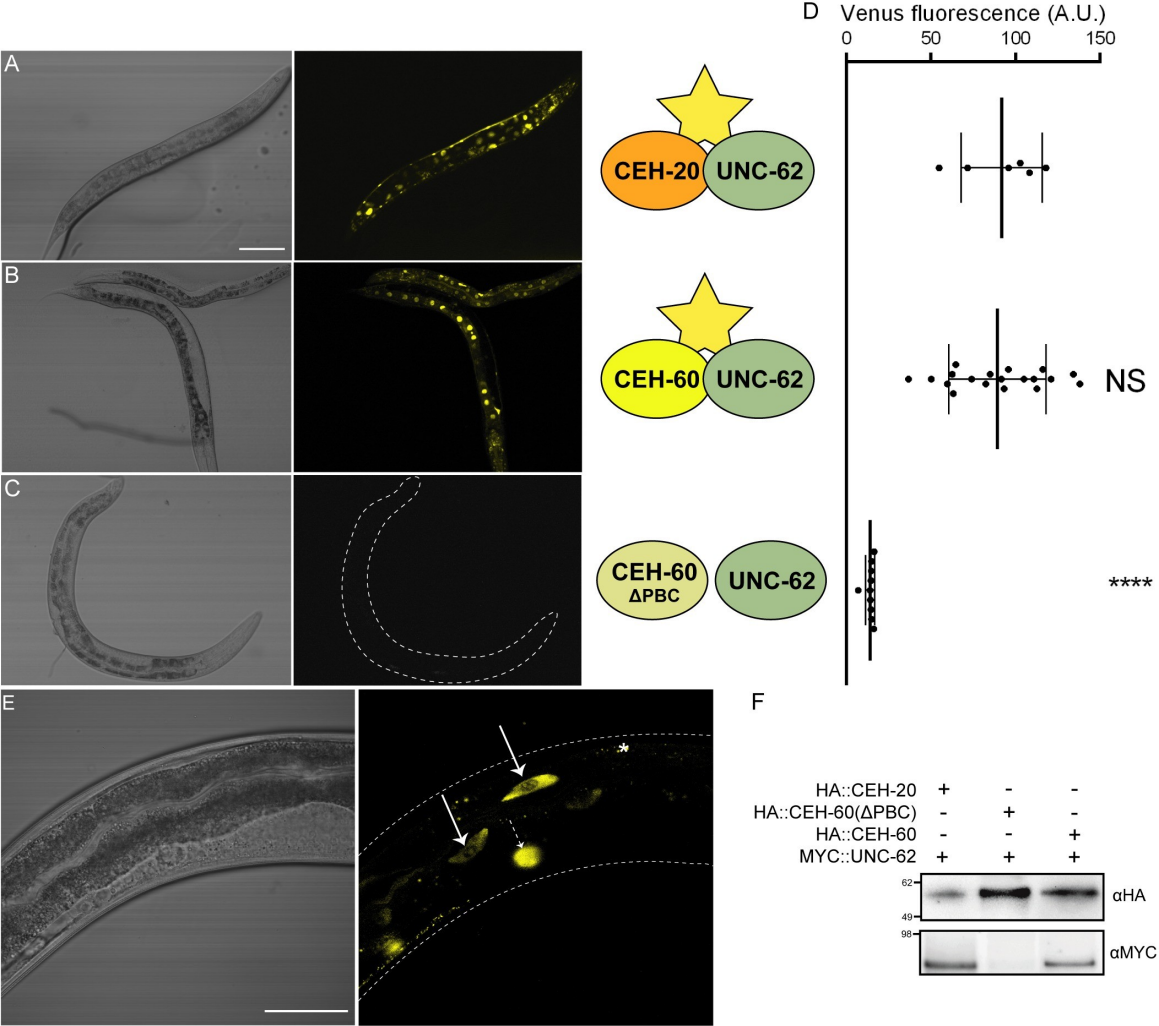
Bimolecular fluorescence shows in vivo interaction between UNC-62 and CEH-60 in the adult intestine. (A) Bright-field and YFP images of animals carrying *hsp-16*.*41p*::*ceh-20*::*VC155* and *hsp-16*.*41p*::*unc-62*::*VN173*. In vivo interaction between CEH-20 and UNC-62 reconstitutes fluorescence ubiquitously after heat shock (33°C, 2 hours), most clearly visible in the intestinal nuclei. (B) Interaction between CEH-60 and UNC-62 reconstitutes fluorescence in animals carrying *hsp-16*.*41p*::*ceh-60*::*VC55* and *hsp-16*.*41p*::*unc-62*::*VN173* after heat shock (33°C, 2 hours). (C) Interaction between CEH-60 and UNC-62 is not observed upon expression of a version of CEH-60 in which the PBC domain is not present, CEH-60(ΔPBC), indicating that this domain is needed for the interaction. (D) Quantification of Venus fluorescence for interaction between UNC-62 and CEH-20, CEH-60, or CEH-60(ΔPBC). One dot represents average fluorescence intensity in 6 intestinal nuclei per animal. ****p < 0.0001. *N* ≥ 6. Underlying data are available in S1 Data. (E) Bright-field and YFP images of animals carrying *ceh-60p*::*ceh-60*::*VC155* and *unc-62p*::*unc-62*::*VN173* transgenes, providing spatiotemporal specificity to the endogenous in vivo interaction of CEH-60 and UNC-62 in the adult intestine (solid arrows). No YFP signal was observed in other tissues besides the intestine. Reflecting the extrachromosomal nature of the transgenes, not all transgenic adult animals showed clear YFP in the intestine. Higher magnifications were used for endogenous BiFC because the YFP signal is much weaker compared to that of induced heat-shock promoters (A-C). Dotted arrow = signal of co-injection marker *unc-122p*::*DsRed*, * = fluorescent gut granule. Scale bar for A, B, and C = 100 μm. Scale bar for D = 50 μm. (F) Western blots detecting HA-tagged CEH-20, HA-tagged CEH-60, HA-tagged CEH-60(ΔPBC), or MYC-tagged UNC-62 in anti-HA immunoprecipitations using anti-HA and anti-MYC antibodies. CEH-20 and CEH-60, but not CEH-60(ΔPBC), co-immunoprecipitate UNC-62. A.U., arbitrary unit; BiFC, bimolecular fluorescence complementation; NS, not significant; YFP, yellow fluorescent protein.

Intestinal expression of *ceh-60* opens up the possibility that CEH-60, in line with what is known for the two other PBC-class proteins of *C*. *elegans* [14,15], exerts its function through direct interaction with the MEIS-class transcription factor UNC-62. To assess whether CEH-60 and UNC-62 interact in vivo, we relied on bimolecular fluorescence complementation (BiFC). We genetically fused each of two halves of a fluorescent reporter protein (i.e., Venus N- and C-terminal parts) to two potentially interacting proteins (i.e., UNC-62 and possible partners). Upon in vivo interaction between the candidates, fluorescence of Venus is reconstituted. To test the capability of the BiFC assay for finding UNC-62 partners, we first probed for interaction between UNC-62 (N-Venus) and CEH-20 (Venus-C), a known genetic interactor of UNC-62 [17]. Using this system under control of a ubiquitously expressed heat shock promoter, we successfully observed in vivo interaction between UNC-62 and CEH-20 (Fig 3A). Similarly, UNC-62 and CEH-60 are clearly able to interact in vivo (Fig 3B). However, when we truncate the PBC domain of CEH-60, which is predicted to interact with UNC-62 [28–31], no signal is observed (Fig 3C). Indeed, Venus signals from CEH-20 or CEH-60 interacting with UNC-62 are significantly higher than the mere noise levels registered for CEH-60(ΔPBC) and UNC-62 (Fig 3D). Our results thus show that CEH-60 interacts with UNC-62 in vivo in a manner that depends on the PBC domain of CEH-60, at least when overexpressed upon heat shock.

To provide spatial information relevant to the endogenous interaction, we cloned the promoters of *unc-62* and *ceh-60* into their respective BiFC expression vectors. The clear Venus signal in the intestinal nuclei of adults (Fig 3E) indicates that endogenous CEH-60 and UNC-62 interact in vivo in the intestine. Using the heat-shock inducible BiFC expression strains, we found that intact CEH-60 co-immunoprecipitates with UNC-62, in contrast to CEH-60 with a truncated PBC domain. This confirms the BiFC observations indicating that CEH-60 and UNC-62 interact and also shows that the PBC domain of CEH-60 is required for this interaction (Fig 3F).

Notably, we did not observe any BiFC interaction in larvae and never observed reporter fluorescence in the pharynx or neurons, where CEH-60 is also present. Thus, the interaction between CEH-60 and UNC-62 spatiotemporally coincides with vitellogenesis. Because yolk protein levels could not be restored in *ceh-60* mutants that are rescued with a PBC domain-truncated variant of CEH-60 (Fig 2), we conclude that the interaction with UNC-62 is necessary for CEH-60 to fulfill its vitellogenesis-regulating function.

### CEH-60 regulates cuticle permeability and morphology

The only paralogs of *ceh-60*, i.e., *ceh-20* and *ceh-40*, code for proteins that interact with UNC-62 to regulate cuticle development through controlling the division of seam cells, the hypodermal cells that synthesize the extracellular cuticle matrix [15]. While loss of functional *ceh-20*, *-40*, or *unc-62* causes seam cell hyperplasia [15], we observed a normal number of seam cells in *ceh-60(lst466)* animals (S3 Fig). This is not entirely unexpected, because we did not observe *ceh-60* expression in any hypodermal cells (Fig 1 and S2 Fig).

**Fig 4:**
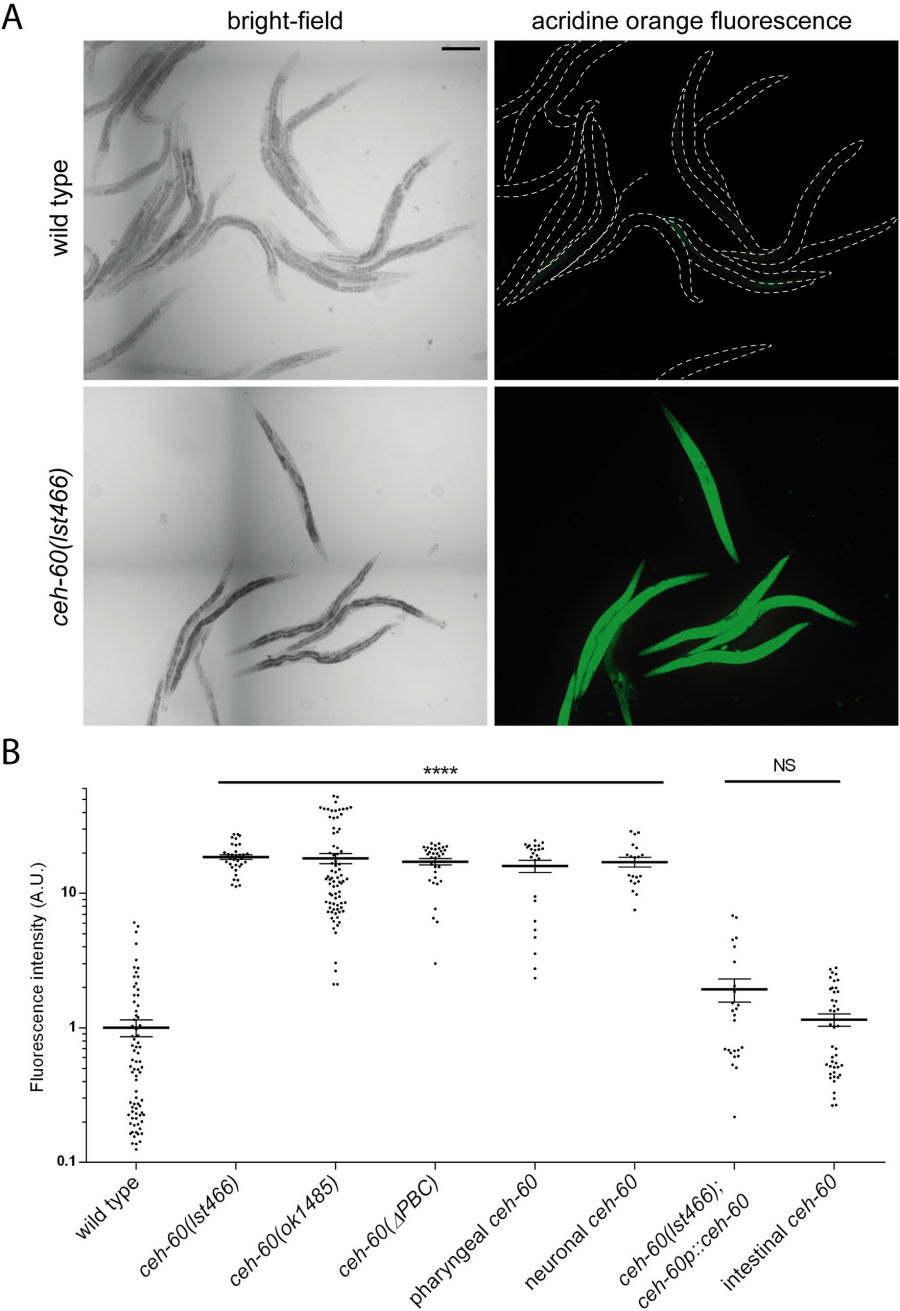
*ceh-60* mutants have a more permeable cuticle. (A) Representative images of acridine orange staining in wild-type and *ceh-60(lst466)* animals. Dotted white lines in wild-type acridine orange images show worm outline. Scale bar = 200 μm. (B) Acridine orange stains *ceh-60* mutants but not wild-type animals. Expressing wild-type *ceh-60* under the control of its own promoter *(ceh-60p*::*ceh-60)* or under an intestinal promoter (*elt-2p*::*ceh-60*) rescues the defect in *ceh-60(lst466)* mutants, but neuronal (*odr-1p*::*ceh-60*), pharyngeal (*myo-2p*::*ceh-60*), or PBC-truncated *(ceh-60p*::*ceh-60(ΔPBC))* expression does not. Fluorescence intensity is shown on a logarithmic scale for clarity. ****p < 0.0001. *N* ≥ 20. Underlying data are available in S1 Data. A.U., arbitrary unit; NS, not significant.

Yet, cuticle collagens were noticeably down-regulated in our differential proteomics data (Table 1), and *ceh-60* mRNA expression cycles, according to the molting cycle, peaked at the transition from molt to early next stage (S8 Fig, [3]). This led us to probe for cuticle integrity by exposing animals to acridine orange, a fluorescent DNA- and RNA-staining dye that is normally blocked by the rigid *C*. *elegans* cuticle [32]. Wild-type animals only show very weak fluorescence after exposure, but the signal of *ceh-60(lst466)* animals is extremely strong (Fig 4A and 4B), indicative of a cuticle highly permeable to this dye. This defect is rescued by expressing CEH-60 endogenously (*ceh-60p*::*ceh-60*) or in the intestine (*elt-2p*::*ceh-60*) of mutant animals, but not by restoring *ceh-60* expression in the AWC neurons (*odr-1p*::*ceh-60*) or pharynx (*myo-2p*::*ceh-60*) (Fig 4B), showing that intestinal action of CEH-60 is needed for creating a cuticle impermeable to this dye. Because *ceh-60(ok1485)* mutants also display this phenotype (Fig 4B), we asked whether the down-regulated collagen proteins observed in our proteomics data (but not in the RNAseq analysis) could in fact be contributors to this hyperpermeability phenotype. RNAi knockdown of either *col-106* or *col-120* did not result in increased permeability (S4 Fig), arguing that these collagens may be down-regulated due to CEH-60 disruption, but their down-regulation in itself is insufficient to create a permeable cuticle.

**Fig 5:**
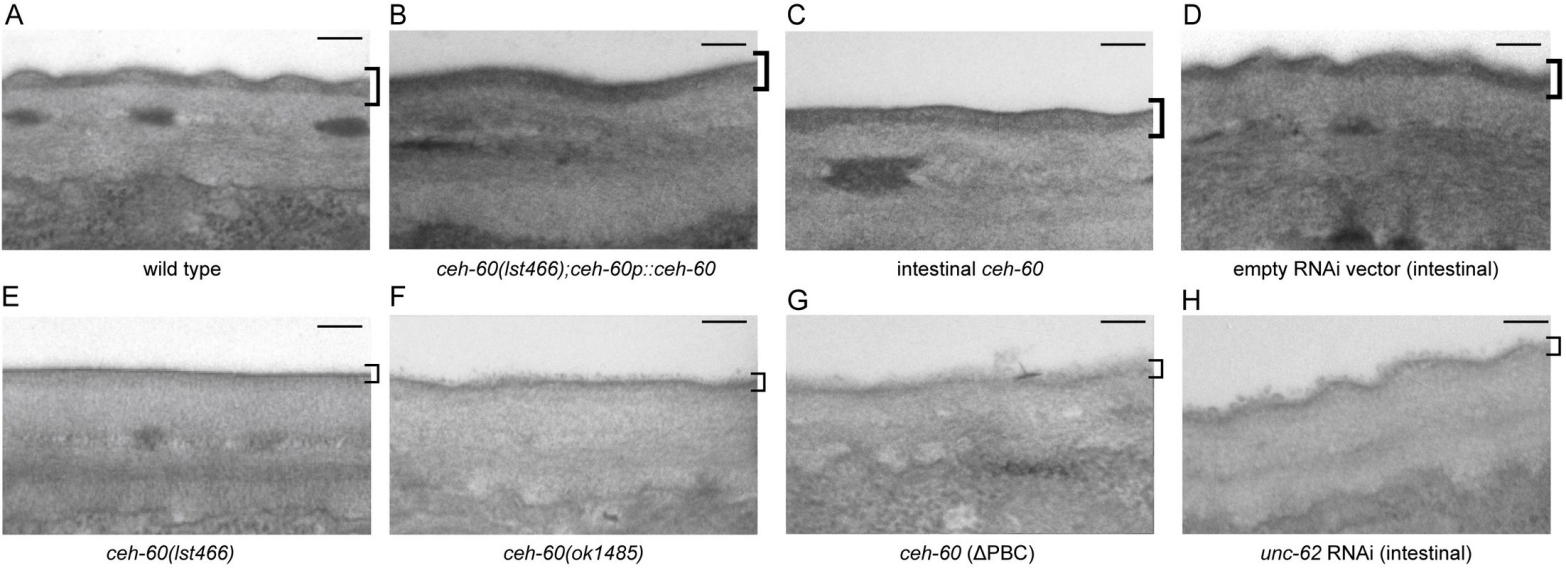
TEM unveils epicuticle problems in *ceh-60* mutants. The epicuticle is visible as a thick electron-dense layer at the outer side of the cuticle in wild-type animals (A), or when *ceh-60(lst466)* mutants are rescued with endogenous (*ceh-60p*::*ceh-60* [B]) or intestinal (*elt-2*::*ceh-60* [C]) *ceh-60* expression. In contrast, the epicuticle is a thin and underdeveloped layer in *ceh-60* mutants (E,F), or when a variant of CEH-60 with truncated PBC-interaction domain is expressed (*ceh-60p*::*ceh-60(ΔPBC)* [G]). Treatment with intestinal *unc-62* RNAi (H) results in an epicuticle that is intermediate between those of empty vector–treated animals (D) and *ceh-60* mutants. Square brackets indicate the epicuticle region in all panels. Scale bar = 100 nm. RNAi, RNA interference; TEM, transmission electron microscopy.

Transmission electron microscopy (TEM) next revealed that whereas wild-type adults show an electron-dense, thick, outer layer of the cuticle, this layer is severely underdeveloped in *ceh-60* mutants, as visible from its smooth and thin surface (Fig 5). While little is known about this lipid-rich epicuticle layer, it is believed to be important for small molecule permeability, including the acridine orange used here [32–35]. In line with the permeability assays described above, intestinal or endogenous, but not neuronal or pharyngeal, expression of *ceh-60* is able to restore a wild type–like epicuticle (Fig 5).

**Fig 6:**
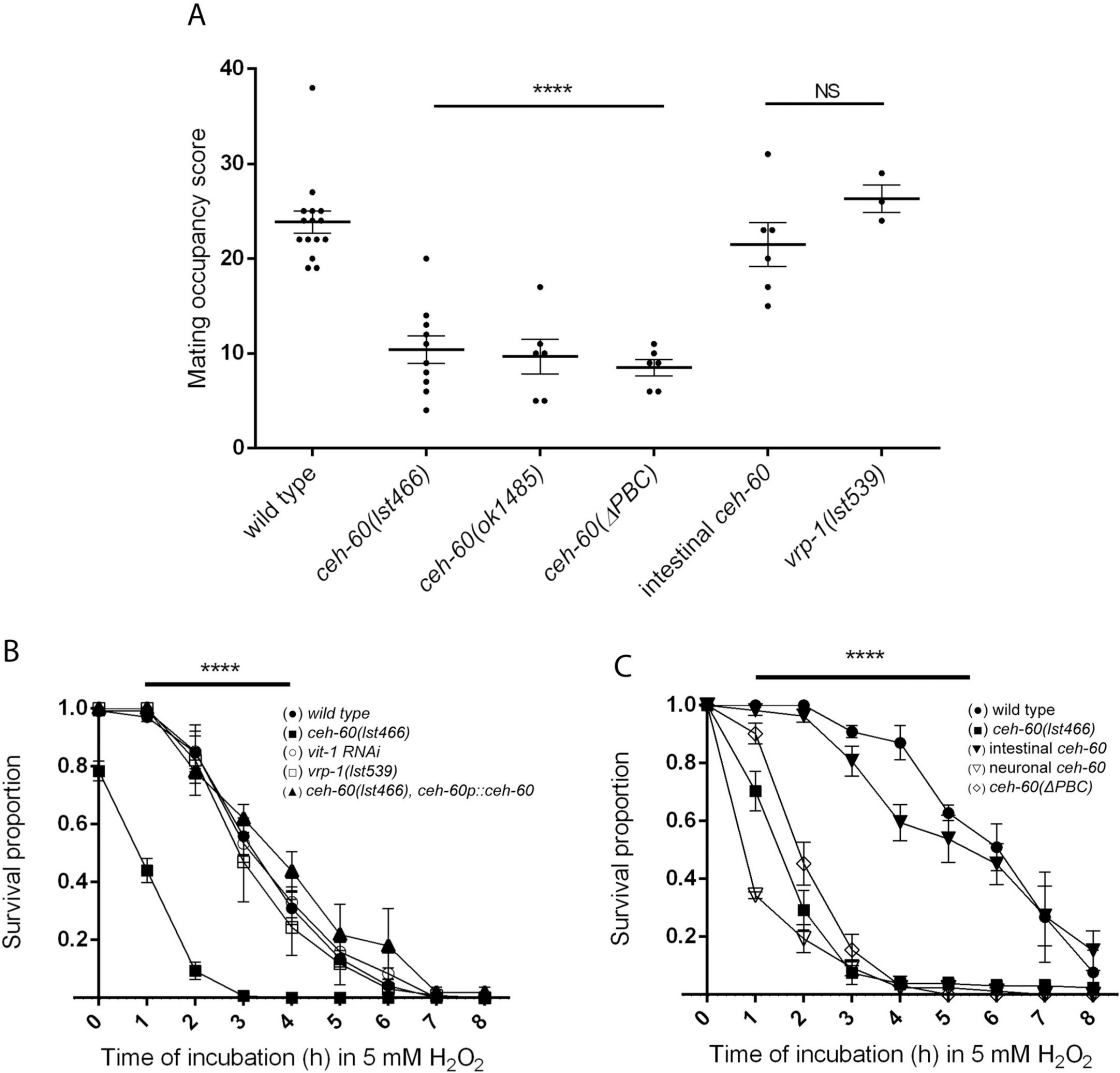
CEH-60 is essential for normal mating contact and survival of oxidative stress. (A) The mating occupancy score of *ceh-60* but not *vrp-1* mutants is lower than wild-type animals, indicating a defect in mating contact that is not caused by lowered yolk protein production. Mating contact deficiency is rescued by expressing *ceh-60* under the control of its own promoter (*ceh-60p*::*ceh-60*) or in the intestine (*elt-2p*::*ceh-60*) of *ceh-60(lst466)* mutant animals, but not when expressing PBC-truncated *ceh-60* (*ceh-60p*::*ceh-60(ΔPBC)*). *N* = 3 for vrp-1(lst539). *N* ≥ 6 for other conditions. (B) Oxidative stress survival as measured by fraction of worms alive during incubation in 5 mM H_2_O_2_ is lower in *ceh-60(lst466)* mutants (■) than in controls (●). The down-regulation of VITs in *vit-1* RNAi treated animals (○) or *vrp-1(lst539)* animals (□) does not cause increased susceptibility to oxidative stress. Expression of *ceh-60* under its own promoter (*ceh-60p*::*ceh-60*) in *ceh-60(lst466)* mutants (▲) is able to rescue stress survival. (C) Oxidative stress survival in *ceh-60(lst466)* animals (■) is rescued by intestinal expression of *ceh-60* (*elt-2p*::*ceh-60*, *▼*) but not by expression of *ceh-60* in the AWC neurons (*odr-1p*::*ceh-60*, ▽) or expression of *ceh-60* with a truncated PBC-interaction domain (*ceh-60p*::*ceh-60(ΔPBC)*, ◇). *N* ≥ 3. Error bars indicate SEM. ***p < 0.001, ****p < 0.0001. Underlying data are available in S1 Data. RNAi, RNA interference; VIT, vitellogenin.

Because the outer surface of *C*. *elegans* is also important for contact during mating [36], we paired mutant and control hermaphrodites with wild-type males. We observed that for *ceh-60* animals, control males clearly struggle to remain in contact, which is not the case for mating with wild-type hermaphrodites or even other vitellogenesis-deficient mutants. Again, this defect can be rescued by expressing CEH-60 endogenously (*ceh-60p*::*ceh-60*) or in the intestine (*elt-2p*::*ceh-60)* of mutant animals (Fig 6A). Consequently, we propose that mating contact defects are not caused by limited vitellogenesis per se, but by a defect in epicuticle structure, as observed by TEM and driven by lack of intestinal CEH-60.

To identify any other defects in the cuticle of *ceh-60* mutants, we stained the cuticle using the lipophilic dye 1,19-dioctadecyl-3,3,39,39-tetramethylindocarbocyanine perchlorate (DiI) but observed no difference in morphology of annuli, alae, or other structures, although staining does appear more intense in *ceh-60* mutants (S5A Fig). Rhodamine-conjugated wheat germ agglutinin (WGA), which probes the surface antigenicity of the cuticle and which does not stain wild-type animals, shows a clear signal in *ceh-60* mutants, indicating that the glycoprotein layer covering the epicuticle is influenced by lack of functional CEH-60 (S5B Fig). To scan for morphological differences in the cortical layer of the cuticle, we observed expression of a *col-19*::*gfp* reporter but found no structural differences between the wild type and *ceh-60* knockdown (S5C Fig). Taken together, permeability assays, TEM micrographs, mating assays, and cuticle stainings show that the deformation in the surface of *ceh-60* animals is limited to the epicuticle and, likely as a consequence, the glycoprotein layer that covers it.

### Cuticle permeability explains susceptibility to exogenous oxidative stress

Increased cuticle permeability to small molecules could affect the resistance of animals to many environmental stressors. We indeed found that upon exposure to reactive oxygen species (ROS; here, 5 mM H_2_O_2_), *ceh-60* mutants perish faster than controls, a defect that can be rescued by expressing *ceh-60* under an endogenous (*ceh-60p)* or an intestinal (*elt-2p)* promoter, and is partially phenocopied by intestinal knockdown of *unc-62* (Fig 6B and S6 Fig). We reasoned that decreased oxidative stress resistance could also be caused by absence of the protective effect of VITs during stress in *ceh-60* instead of by an increased permeation of exogenous ROS. The former has been observed in other animals [37] and in *C*. *elegans* when preceded by infection [38]. However, *vrp-1* mutants with similarly low yolk levels as *ceh-60* mutants [3] and animals treated with RNAi against *vit-1* all displayed wild-type oxidative stress susceptibility. Hence, stress sensitivity is not caused by VIT deficiency but by a distinct effect of CEH-60. To show that increased permeation of stressors in *ceh-60* mutants is not limited to hydrogen peroxide or oxidative stress per se, we confirmed these findings with another small molecule, the inhibitor of cytochrome C oxidase sodium azide (S7 Fig).

**Fig 7:**
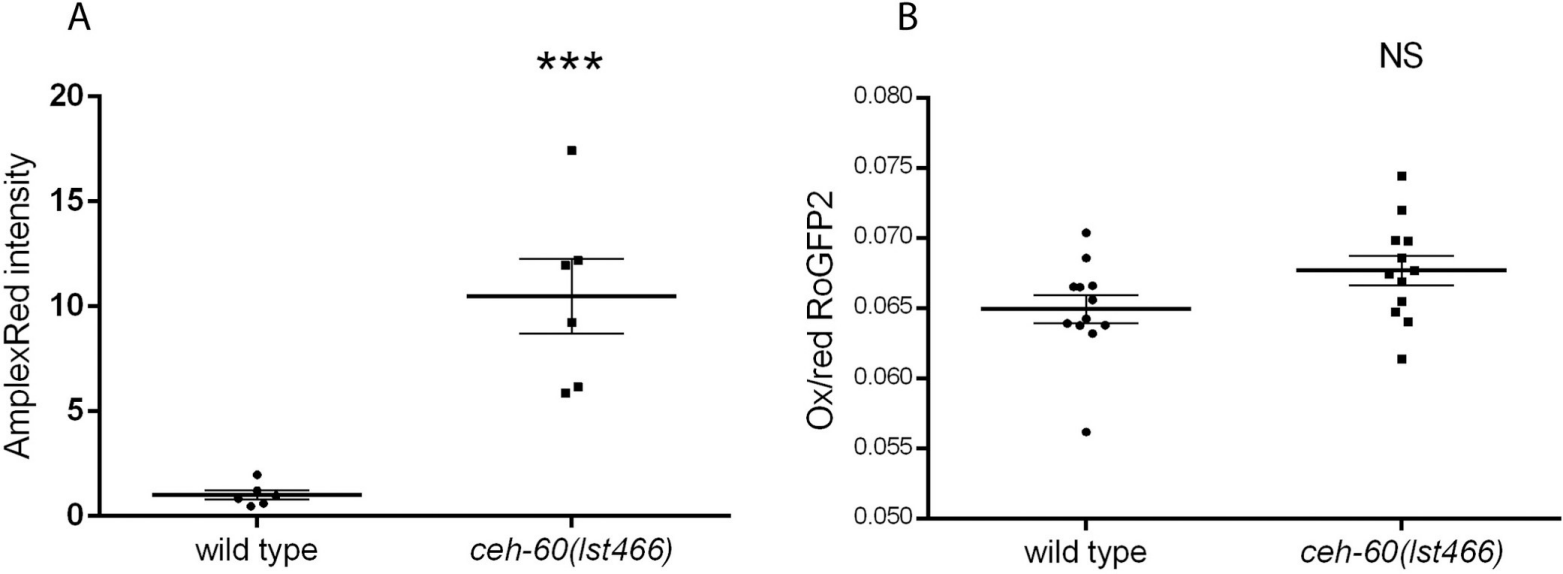
Dysfunctional CEH-60 causes a cuticle that is hyperpermeable to endogenous ROS. (A) In supernatants collected from *ceh-60(lst466)* animals, the intensity of Amplex Red absorbance is much higher than in that of wild-type animals. Values are normalized to control. *N* = 6. (B) Endogenous ROS production measured with the in vivo genetic ROS sensor RoGFP2, indicated as the ratio of oxidized over reduced GFP, is the same in *ceh-60* and wild-type animals. Error bars, SEM; ***p < 0.001. *N* = 12. Underlying data are available in S1 Data. GFP, green fluorescent protein; NS, not significant; Ox/red, ratio of oxidized over reduced GFP; RoGFP2, reduction-oxidation sensitive green fluorescent protein; ROS, reactive oxygen species.

Increased permeation of exogenous stressors into *ceh-60* animals due to a faulty cuticle also predicts that endogenously produced ROS should diffuse into the environment at higher rates in mutants. Indeed, biogenic ROS permeation from the animal into the environment, as measured with the dye Amplex Red [39], is much higher in *ceh-60(lst466)* animals (Fig 7A). This signal could alternatively be caused by a higher internal ROS production of mutants. Arguing against this is the observation that *ceh-60* animals expressing a redox-sensitive version of GFP, RoGFP2 [40], showed a normal balance of oxidized over reduced protein, indicating that their redox state is unaltered compared with wild type (Fig 7B). When challenged with an exogenous oxidative stress shock (i.e., addition of a nonlethal dose of H_2_O_2_), *ceh-60* mutant animals, however, respond with an exacerbated oxidized versus reduced protein status in comparison with wild-type animals (S9 Fig). Together, these findings show that dysfunction of CEH-60 does not alter exogenous stress resistance through a baseline imbalance of internal redox state but through increased permeability.

### Hyperpermeability depends on intestinal interaction between CEH-60 and UNC-62

While regulation of vitellogenesis by CEH-60 depends on interaction with UNC-62, and while a developmental signal connecting the hypodermis to the intestine is able to activate UNC-62 [11], it remains unclear if the permeability controlled by CEH-60 is similarly regulated by intestinal interaction with UNC-62. A PBC domain–truncated version of CEH-60 (defective in UNC-62 interaction, cf. supra) could not rescue vitellogenesis, hyperpermeability, epicuticle morphology, mating contact, stress resistance, or sodium azide sensitivity, while expression of *ceh-60* in the intestine alone (*elt-2p*) suffices to restore these phenotypes to wild-type levels (Figs 2, 4B, 5C, 6A and 6C and S7 Fig). Hence, the interaction between CEH-60 and UNC-62 drives both vitellogenesis and cuticle permeability in a way that originates from the primary lipid-metabolizing organ in *C*. *elegans*, the intestine.

## Discussion

Studying the genetics of animal reproduction aids in understanding how species integrate age-, sex-, and environmentally relevant signals to maximize their reproductive fitness. From a fundamental, conceptual perspective, the successful production of high-quality gametes is thus a manifestation of an animal’s ability to manage a multi-tissue, multi-input type of complexity towards a very focused effect. *C*. *elegans* is a small and fast-reproducing model organism in which the synthesis of enormous amounts of yolk proteins provides an ideal readout to address such questions and unveil fundamental genetics of reproduction [3,6,9,11,41].

In earlier work, we identified the three amino acid loop extension (TALE) PBC-class Hox cofactor CEH-60 as a potential molecular gateway to kick-starting yolk protein production in *C*. *elegans* [3]. Its then-reported expression in a single neuron pair—here identified as the olfactory AWC neurons (S2A Fig)—incentivized us to unveil CEH-60’s precise role in organismal control of intestinal yolk production. We found that CEH-60, in line with what is known for other TALE proteins [15,42–46], influences a range of physiological processes, including cuticle permeability, stress resistance, and the aforementioned vitellogenesis. Our finding that *ceh-60* is additionally expressed in the pharynx and L4/adult intestine (Fig 1, S2B and S2C Fig) is corroborated by available genome-wide tissue-specific profiling data indeed revealing *ceh-60* transcripts in amphid neurons, intestine, and pharynx [47,48]. We pinpointed the intestine as the site of CEH-60’s vitellogenesis-regulating function, a finding also recently reported by Robert Dowen and thus strengthening our conclusion [12]. Because CEH-60 is not dedicated to yolk production only, we asked how it might be recruited to this function.

Hox cofactors, and particularly TALE proteins, are able to regulate a myriad of physiological processes, not only those limited to development or even those requiring the action of a Hox protein [45,49–52]. PBC-class proteins interact with MEIS-class transcription factors, of which there is only one representative in *C*. *elegans*: UNC-62. The PBX/MEIS interaction is widely conserved and well documented in vertebrates (reviewed in [53]). CEH-60/PBX and UNC-62/MEIS have also been studied in *Drosophila melanogaster*, in which they are named extradenticle (EXD) and homothorax (HTH) [28–30], indicative of an evolutionary conservation in the protostome lineage. In *C*. *elegans*, the only other PBC-class proteins, CEH-20 and CEH-40, interact with UNC-62 to function in hypodermal and neuronal development [14,15]. UNC-62 is known to additionally function in the *C*. *elegans* intestine as an activator of vitellogenesis, without knowledge of a corresponding PBX-like cofactor. Here, we identified this elusive vitellogenesis-regulating partner of UNC-62 as CEH-60 and showed that its function depends on the ability to interact with UNC-62 in the intestine (Figs 2 and 3). Via independent methods, a recent study by Robert Dowen came to the same conclusions [12]. In his work, Dowen showed that CEH-60 shares specific DNA-binding sites with UNC-62, including the promoters of the *vit* genes, and that UNC-62 and CEH-60 co-immunoprecipitate. Dowen illustrates that the CEH-60::UNC-62 heterodimer acts as a metabolic switch to mediate organismal homeostasis from the intestine. It does so by activating reproductive programs, such as vitellogenesis, and repressing somatic maintenance programs, such as innate immunity and life span. He additionally shows that at least one other transcription factor, PQM-1, is involved in co-regulating CEH-60::UNC-62 action. These findings, together with our own work, establish CEH-60/PBX as a linchpin of metabolism and development. The study by Dowen supports the main conclusions from our own work, which independently shows that CEH-60 and UNC-62 act together as powerful regulators of the mobilization of the main intestinal resources: yolk proteins and lipids.

Our study adds further depth by spatiotemporally defining the site of the interaction between CEH-60 and UNC-62 in the intestinal nuclei of adult animals (Fig 3), coinciding with the time and place of vitellogenesis. Furthermore, we show that the PBC domain is essential for interaction with UNC-62, activation of vitellogenesis, and the building of an impermeable cuticle (Figs 2C, 4B, 5 and 6C). While we cannot rule out the possibility that the PBC domain of CEH-60 may support DNA binding or other protein interactions besides the one with UNC-62, our PBC-truncated version of CEH-60 still includes the homeodomain and 16-residue C-terminal domain necessary for DNA binding in PBC-class TALE proteins [13], and no other PBC-dependent interactions besides those with UNC-62/MEIS/PBX regulating protein (PREP) are known [53].

While *ceh-60* expression increases enormously at the start of vitellogenesis (S8 Fig, [3]), our work provides plenty of evidence for other functions, most notably in epicuticle structure and permeability (Figs 4–6). Similar to our observations for *ceh-60* (Figs 4 and 5), other mutants with compromised (epi)cuticle integrity are more permeable to several compounds, including exogenous ROS or fluorescent dyes [34,54], and have difficulty staying in contact with a partner during mating [36]. Many of the known epicuticle-disrupting genes code for glycosyltransferase enzymes or other direct constituents of the (epi)cuticle, while CEH-60 is, to our knowledge, the first transcription factor to affect cuticle permeability.

It is not likely that CEH-60 works directly in the hypodermal cells, as no expression in those cells was observed. We cannot rule out the possibility that hypodermal expression may be too weak to detect, as epidermal and seam cells form large syncytia [35] and the CEH-60::GFP signal may be too diffuse. Still, CEH-60’s interaction partner, UNC-62, is known to be involved in cuticle synthesis. Among genes differentially expressed after *unc-62* RNAi, 41 of 115 were collagens, and UNC-62 interacts with several transcription factors that function in the proper development of hypodermal cells and/or cuticle, corroborating the role of CEH-60’s interaction partner in cuticle development [16]. However, unlike CEH-20, CEH-40, and UNC-62, CEH-60 does not obviously affect the development of the seam cells (S3 Fig) that are likely responsible for synthesis of the cuticle [55].

Instead, we here find that the intestinal function of CEH-60 is not limited to the regulation of vitellogenesis, but includes the formation of an impermeable cuticle. While our reporters revealed no or very little intestinal expression before the L4 stage (Fig 1), single-cell sequencing data suggest CEH-60 transcripts to be present in the larval (at least L2) intestine [47]. We also observed oscillating *ceh-60* expression during development, in synchrony with the molting cycle (S8 Fig). These whole-mount data may reflect the cyclic need for epicuticle building but lack finite spatial information. To our knowledge, synthesis of the epicuticle has not been studied in detail in *C*. *elegans*, but it has been suggested that the pharyngeal gland cells secrete the surface coat of other nematode species [56,57]. In *C*. *elegans*, defects in pharyngeal muscle structure have been shown to lead to deformed pharyngeal gland cells [58], which might also be the case in *ceh-60* mutants. Pharyngeal *ceh-60* expression, however, could not restore cuticle impermeability in mutants (Fig 4B), indicating that this is an unlikely scenario for CEH-60.

Instead, we found that it is intestinal CEH-60 that affects the morphology and permeability of the lipid-rich epicuticle (Figs 4B and 5C). The obvious hypothesis would be that it does so through its influence on lipid homeostasis. The lower abundance of some lipid-metabolizing enzymes observed in *ceh-60* mutants (Table 1) may simply reflect the inability of the intestine to mobilize lipids in response to a hypodermal need. Expression of *ceh-60* is likely not just a signal to start the production of yolk proteins, but a more general cue to start mobilization of lipids to either end: i.e., yolk production or epicuticle assembly.

Supporting the hypothesis of CEH-60 being involved in intestine-to-hypodermis lipid mobilization is the discovery of hypodermal micro-RNAs (miRNAs) that, in *C*. *elegans*, signal the start of intestinal mobilization of fat for yolk protein production in a manner that ultimately depends on intestinal activation of UNC-62 [11]. Why the signal originates in the hypodermis and how it is activated is currently unknown. It is, however, clear that in cuticle construction as well as in yolk production, there must be coordinated communication between the hypodermis and the intestine and that the intestinal interaction of CEH-60/PBX and UNC-62/MEIS is a central target in these processes. This knowledge provides a framework to continue adding to our understanding of the multi-tissue, multi-input complexity of reproductive control.

The increased cuticle permeability may add to the appeal of *ceh-60* mutants as a tool for drug screening. While *C*. *elegans* is used for, e.g., toxicity screens [59–61], its rigid and impermeable cuticle has been a noted disadvantage of this otherwise efficient and low-cost model organism [54,62,63]. A number of *C*. *elegans bus* (*b*acterially *u*n-*s*wollen) mutants have been proposed for toxicity screening precisely because of their increased cuticle permeability, but their general fitness is far from ideal, which questions practical applicability [32]. Mutants for *ceh-60* could provide an alternative to wild-type nematodes or *bus* mutants, as their overall fitness, as measured by life span and reproductive potential, does not seem to be impaired [3,12]. A note of caution is due, however, because the pharyngeal and neuronal roles of CEH-60 are yet to be described. In our hands, *ceh-60* mutants are easily discernible from wild-type animals but are overall healthier than *bus* mutants.

In other organisms, PBX and MEIS, as well the interaction between the two, are conserved [28–30,53]. They have been studied mostly with regard to neuronal development [46,64] and body plan patterning (reviewed in [65]), although evidence is mounting for the involvement of TALE proteins (PBX, MEIS, and PREP) in lipid homeostasis and metabolism (reviewed in [66]). It is well established that the ancient co-operation between PBX and MEIS reaches beyond their initially discovered roles as mere Hox cofactors. The functions of CEH-60/PBX and UNC-62/MEIS in *C*. *elegans* vitellogenesis and epicuticle formation provide the groundwork to expand on their scarcely investigated involvement in lipid homeostasis in other organisms as well.

## Materials and methods

### Strains and plasmids

*C*. *elegans* strains were grown under standard conditions [67], fed with *Escherichia coli* OP50, and raised at 20°C unless stated otherwise. For details on strain names and genotypes, see Table 2.

**Table 2:** List of *C*. *elegans* strains used in this study, including genotype and source.

Strain name	Genotype	Source/reference
PY2417	*oyIs44 [odr-1p*::*rfp; lin-15(+)]*	*Caenorhabditis* Genetics Center, University of Minnesota, MN, United States
JR667	*wIs51 [SCMp*::*gfp; unc-119(+)]*	*Caenorhabditis* Genetics Center, University of Minnesota, MN, US
TJ356	*zIs356 [daf-16p*::*daf-16a/b*::*gfp; rol-6(su1006)]*	*Caenorhabditis* Genetics Center, University of Minnesota, MN, US
TP12	*kaIs12[col-19*::*GFP]*	*Caenorhabditis* Genetics Center, University of Minnesota, MN, US
JV10	*jrIs10 [unc-119(+) rps-0p*::*roGFP2-Orp1]*	Gift of Professor B. Braeckman, Ghent University, Belgium [40]
MGH167	*sid-1(qt9); alxIs9 [vha-6p*::*sid-1*::*SL2*::*gfp]*	Gift of Professor B. Braeckman, Ghent University, Belgium
UL2612	*ceh-60p*::*gfp*	Gift of Professor I. A. Hope, University of Leeds, Leeds, United Kingdom [19]
DLS357	*ceh-60(ok1485) X*	Gift of Professor R. Dowen, University of North Carolina at Chapel Hill, NC, US [12]
LSC897	*ceh-60(lst466) X*	[3]
LSC902	*vrp-1(lst539) IV*	[3]
LSC1491	*lstEx790 [ceh-60p*::*ceh-60*::*gfp; unc-122p*::*DsRed] oyIs44 [odr-1p*::*rfp; lin-15(+)]*	this study
LSC1487	*lstEx790 [ceh-60p*::*ceh-60*::*gfp; unc-122p*::*DsRed]*	this study
LSC1501	*ceh-60(lst466) X; lstEx855 [odr-1p*::*ceh-60; unc-122p*::*DsRed]*	this study
LSC1798	*ceh-60(lst466) X; lstEx950 [ceh-60p*::*ceh-60*::*SL2*:: *gfp*::*unc-54 3′ UTR; unc-122p*::*DsRed]*	this study
LSC1595	*ceh-60(lst466) X; jrIs10 [unc-119(+); rps-0p*::*roGFP2-Orp1]*	this study
LSC1684	*ceh-60(lst466) X; lstEx909 [ceh-60p*::*ceh-60*:: *ceh-60 3´ UTR; unc-122p*::*DsRed]*	this study
LSC1814	*lstEx956 [hsp-16*.*41p*::*MYC*::*unc-62*::*VN173*::*unc-54 3′ UTR; hsp-16*.*41p*::*HA*::*ceh-20*::*VC155*::*unc-54 3′ UTR; unc122p*:: *DsRed]*	this study
LSC1815	*lstEx957 [hsp-16*.*41p*::*MYC*::*unc-62*::*VN173*::*unc-54 3′ UTR; hsp-16*.*41p*::*HA*::*ceh-60*::*VC155*::*unc-54 3′ UTR; unc122p*:: *DsRed]*	this study
LSC1816	*lstEx958 [hsp-16*.*41p*::*MYC*::*unc-62*::*VN173*::*unc-54 3′ UTR; hsp-16*.*41p*::*HA*::*ceh-60(lst466)*::*VC155*::*unc-54 3′ UTR; unc122p*:: *DsRed]*	this study
LSC1817	*lstEx959 [hsp-16*.*41p*::*MYC*::*unc-62*::*VN173*::*unc-54 3′ UTR; hsp-16*.*41p*::*HA*::*ceh-60(ΔPBC)*::*VC155*::*unc-54 3′ UTR; unc122p*:: *DsRed]*	this study
LSC1831	*lstEx1022 [unc-62p*::*MYC*::*unc-62*::*VN173*::*unc-54 3′ UTR; ceh-60p*::*HA*::*ceh-60*::*VC155*::*unc-54 3′ UTR; unc-122p*::*DsRed]*	this study
LSC1832	*ceh-60(lst466) X; wIs51 [SCMp*::*gfp; unc-119(+)]*	this study
LSC1833	*ceh-60(lst466) X; lstEx1023 [elt-2p*::*ceh-60*:: *unc-54 3′ UTR; unc-122p*::*DsRed]*	this study
LSC1834	*ceh-60(lst466) X; lstEx1024 [ceh-60p*::*ceh-60(ΔPBC)*::*unc-54 3′ UTR; unc-122p*::*DsRed]*	this study
LSC1842	*ceh-60(lst466) X; lstEx1031 [myo-2p*::*ceh-60*::*unc-54 3′ UTR*, *unc-122p*::*DsRed)]*	this study

### Differential proteomics and data analysis

Six populations of approximately 3,000 day 1 adult animals were sampled per condition. Samples were labeled with a TMTsixplex isobaric labeling set (Thermo Fisher Scientific, Waltham, MA) according to the manufacturer’s protocol. Worms were rinsed off plates in M9 buffer (3.0 g KH_2_PO_4_, 6.0 g Na_2_HPO_4_, 0.5 g NaCl, 1.0 g NH_4_Cl in 1 L H_2_O), washed three times, and lysed using RIPA buffer containing protease and phosphatase inhibitor. Protein homogenate was then sonicated for 30 seconds on ice and centrifuged at 14,000*g* for 15 minutes. Protein concentration was determined using a BCA assay (Thermo Fisher Scientific, Waltham, MA), and 100 μg of protein extract per sample was prepared for protein digestion as noted in the manufacturer’s manual and digested with 2.5 μL trypsin per sample overnight at 37°C. Peptide digests were labeled with TMT Label Reagent, after which the reaction was quenched with 8 μL 5% hydroxylamine. Pooled samples for each set comprising three wild-type and three mutant populations were prepared in a 1:1:1:1:1:1 protein concentration ratio and stored at −80°C. Each pooled sample was separated into 10 fractions. The sample was injected into the UPLC reverse phase system (Acquity, Waters, Milford, MA). Peptides were separated in a C18 Column (XBridge Peptide BEH, 130 Å, 5 μm, 2.1 × 50 mm). The peptides were eluted at a flow rate of 1.5 mL/minutes using a 14-minute linear gradient as follows: 0−9.5 minutes, 60% mobile phase B (MP B); 9.5−10 minutes, 98% MP B. Mobile phase A (MP A): 98% H2O, 2% acetonitrile, and 0.1% formic acid; MP B: 98% acetonitrile, 2% H_2_O, and 0.1% formic acid, but with pH adjusted to 2 by the addition of formic acid. Eluate was collected in 10 fractions. The collected fractions were vacuum dried and each fraction was resuspended in 25 μL of 98% H_2_O: acetonitrile: formic acid (98:2:0.1) solution for injection on the LC-MS. LC-MS analysis was performed on an Eksigent nanoAcquity LC-Ultra system (Waters, Milford, MA) connected to a LTQ Velos Orbitrap mass spectrometer (Thermo Fisher Scientific, Waltham, MA) through a flex nano ESI source (Thermo Fisher scientific, Waltham, MA). The equivalent of 1 μg of total protein of the digested sample was dissolved in 10 μL of 2% acetonitrile in HPLC-grade water. This sample was loaded on the trapping column (Pepmap C18 300 μm × 20 mm, Dionex, Thermo Fisher Scientific, Waltham, MA) with an isocratic flow of 2% acetonitrile in water with 0.1% formic acid at a flow rate of 5 μL/minute. After 2 minutes, the column-switching valve was switched, placing the pre-column online with the analytical capillary column, a Pepmap C18, 3 μm, 75 μm × 150 mm nano column (Dionex, Thermo Fisher Scientific, Waltham, MA). Separation was conducted using a linear gradient from 2% acetonitrile in water, 0.1% formic acid to 40% acetonitrile in water, 0.1% formic acid in 100 minutes. The flow rate was set at 400 nL/minute. The LTQ Orbitrap Velos was set up in a data-dependent MS/MS mode in which a full scan spectrum (350–2,000 m/z, resolution 60,000) was generated. Full scan spectra were followed by a maximum of five dual collision-induced dissociation (CID)/high-energy collision-induced dissociation (HCD) tandem mass spectra. We applied a dynamic exclusion time of 45 seconds.

Mixed HCD/CID spectra were analyzed in MaxQuant 1.6.3.4 (https://www.maxquant.org) [68] using 6-plex TMT as internal labels with a reporter mass tolerance of 0.003 Da, Oxidation(M) and Acetyl as variable modifications, and carbamidomethyl(C) as fixed modification, with a maximum of 5 modifications per peptide. Default orbitrap instrument settings were used and identified peptides were mapped to the UniProt *C*. *elegans* reference proteome (https://www.uniprot.org/proteomes/UP000001940). All other global parameters were used as default, except that match between runs was enabled. Data analysis and normalization was performed using R 3.5.1 (https://www.r-project.org) and Perseus 1.6.5.0 (https://maxquant.net/perseus) [69] to filter out peptides with valid measurement for at least 4 out of 12 channels and without all 6 of one 6-plex’s channels being empty. Proteins were only retained for analysis when at least 2 unique peptides were identified. In total, we identified 7,828 peptides corresponding to 1,749 proteins. Data normalization was performed as described in [70] using internal reference sampling and correcting for sample loading. Statistical significance was computed using a two-way ANOVA with Holm-Sidak multiple comparison correction. The mass spectrometry proteomics data have been deposited to the ProteomeXchange Consortium via the PRIDE [71] partner repository with the dataset identifier PXD013584.

### RNAi experiments

RNAi clones were obtained from the Vidal RNAi library, with the exception of the *col-120* clone, which was obtained from the Ahringer library. All clones were sequence-verified before use. RNAi strains were grown in LB medium containing 100 μg/mL ampicillin and spread on nematode growth medium (NGM) plates supplemented with 50 μg/mL ampicillin and 1 mM isopropyl-b-D-thiogalactoside (IPTG, Sigma-Aldrich, St. Louis, MO). Animals were reared for at least two generations on the specified RNAi clone before conducting experiments.

### Oil-red-O staining

Oil-red-O staining was performed as described in [72]. For each condition, at least 20 animals of the desired age were fixed for 1 hour in Modified Ruvkun’s Witches Brew (80 mM KCl, 20 mM NaCl, 10 mM EGTA, 5 mM spermidine, 15 mM Pipes [pH 7.4], and 25% [v/v] methanol, also containing 2% [w/v] formaldehyde). Afterwards, animals were washed in M9 physiological buffer solution and incubated for 15 minutes in 60% (v/v) isopropanol, followed by overnight incubation in the same solution containing 0.3% Oil-Red-O (Abcam, UK). Stained worms were washed with M9 buffer and imaged using a Axio Imager Z1 microscope (Zeiss, Germany). As per [73], red color intensity was quantified in a circular region of the same size in each animal, as marked in S1 Fig. Stain intensity was quantified in at least 5 animals per condition per time point. Data were analyzed using a two-way ANOVA with a Tukey post hoc test.

### Localization: Cloning and microscopy

Localization constructs were created by cloning a promoter region of 3,464 bp 5′ to the start of the *ceh-60* gene and *ceh-60* cDNA into a modified pSM vector carrying a GFP reporter sequence preceded by an SL2 *trans*-splicing site (kindly provided by C. Bargmann, Rockefeller University, New York, NY). The promoter and cDNA sequences were inserted right before the SL2 site using NEBuilder HiFi DNA Assembly Master Mix (New England BioLabs, Ipswich, MA) and transformed into DH5-alpha competent cells. Purified plasmid DNA was microinjected into the syncytial gonad of young adult worms at 25 ng/μL together with a co-injection marker *unc-122p*::*DsRed* at 50 ng/μL. Localization strains were mounted on 2% agarose pads, anesthetized with 1 mM tetramisole, and visualized with a confocal FluoView1000 microscope (Olympus, Japan) or a DM6 B microscope (Leica, Germany).

### BiFC

BiFC was performed as described in [74]. Plasmids pCE-BiFC-VN173 and pCE-BiFC-VC153 were provided by Addgene (deposited there by the lab of Chang-Deng Hu). *unc-62* and *ceh-20* or *ceh-60* cDNA were cloned into pCE-BiFC-VN173 and pCE-BiFC-VC153, respectively, behind a heat-shock promoter using NEBuilder HiFi DNA Assembly Master Mix (New England BioLabs, Ipswich, MA) and transformed into DH5-alpha competent cells. Purified plasmid DNA of interacting BiFC partners was microinjected into *C*. *elegans* adults together with a co-injection marker, *unc-122p*::*DsRed*, as described above. The endogenous promoters of *unc-62* and *ceh-60* were subsequently cloned into the resulting vectors using the same cloning methods, replacing the heat-shock promoters to provide tissue specificity to the assay.

Prior to screening BiFC-transgenic lines containing a heat-shock promoter, day 1 adult animals were heat-shocked for 2 hours at 33°C. Fluorescent images were taken 5–6 hours after heat shock on a confocal FluoView FV1000 microscope (Olympus, Japan) equipped with an EYFP fluorescence filter. Animals were mounted on 2% agarose pads and anesthetized with 1 mM tetramisole. The fluorescence signal was quantified by measuring the average pixel intensity in 6 intestinal nuclei of at least 6 animals per condition in Fiji [75,76]. No heat shock treatment was administered to animals carrying endogenous promoters.

### Co-immunoprecipitation and western blotting

For co-immunoprecipitation, at least 8 fully grown 90-mm NGM plates of each strain were used. Animals were rinsed off plates and washed in M9 buffer until all bacteria were removed. Per sample, a 500-μL worm pellet topped up with 1 mL of ice-cold lysis buffer (25 mM Tris-HCl [pH 7.5], 100 mM NaCl, 1 mM EDTA, 0.5% NP-40, 1 mM Na_3_VO_4_, 10 mM NaF, 1× complete protease inhibitor cocktail tablets) was snap-frozen in liquid nitrogen before sonicating thrice for 10 seconds with a SLPe Sonifier (Branson, Danbury, CT), snap-freezing, and thawing in room temperature water in between sonication steps. Worm lysates were incubated while agitating at 4°C for 30 minutes and spun down for 30 minutes at 4°C at 13,000 rpm, after which protein concentration was determined with a standard BCA assay. For co-immunoprecipitation, Pierce Anti-HA Agarose beads (Thermo Fisher Scientific, Waltham, MA) were equilibrated according to the manufacturer’s instructions. Beads were incubated at 4°C overnight, with at least 4 mg of worm protein in 1 mL lysis buffer. After washing beads 5 times with lysis buffer, bead-bound proteins were eluted with XT sample buffer and boiled at 98°C for 15 minutes, followed by western blot using anti-HA high affinity (Sigma-Aldrich, St. Louis, MO) and Myc Tag monoclonal (Thermo Fisher Scientific, Waltham, MA) antibodies as primary antibodies and polyclonal Rabbit-Anti-Rat IgG/HRP (Agilent, Santa Clara, CA) or polyclonal Goat-anti-Mouse IgG/HRP (Agilent, Santa Clara, CA) as secondary antibodies. Bands were visualized on a Bio-Rad Gel-Doc following staining in SuperSignal West Dura (Thermo Fisher Scientific, Waltham, MA) according to the manufacturer’s instructions.

### Yolk protein analysis

Coomassie staining of worm protein extracts using SDS-PAGE was performed as described in [3,27]. Synchronous worm populations were grown until day 3 of adulthood, after which 50 animals per sample were picked into 15 μL of M9 buffer. A total of 15 μL of Laemmli sample buffer containing β-mercaptoethanol was added. Samples were incubated for 15 minutes at 70°C, centrifuged at max speed for 5 minutes, and incubated at 95°C for 5 minutes. A total of 15 μL of each sample was loaded on a 4%–12% bis-tris Criterion XT precast polyacrylamide gel (Bio-Rad, Hercules, CA) using XT MOPS as a running buffer. Gels were stained with Coomassie Brilliant Blue and destained with a 40% methanol, 10% acetic acid solution. Gel images were taken with a Bio-Rad Gel Doc (Bio-Rad, Hercules, CA). Identification of YP170, YP115, and YP88 bands is based on [7,27]. Quantification of yolk protein abundance was done by normalizing to total protein content in a lane using ImageLab 6.0. At least three populations were assayed for each condition. Data were analyzed using a one-way ANOVA with a Tukey post hoc test.

### Cuticle permeability and paralysis sensitivity assays

Cuticle permeability assays using acridine orange were performed as described in [32]. In brief, day 1 adult animals were washed off plates, stained with 5 μg/mL acridine orange in M9 buffer for 15 minutes with gentle agitation, followed by three wash steps in M9 buffer. Worms were then mounted on 2% agarose pads and anesthetized with 1 mM tetramisole. Worms were imaged using a DM6 B microscope (Leica, Germany) with GFP filter set. Average fluorescence intensity for at least 20 worms per condition was quantified with Fiji [76] and analyzed using a one-way ANOVA with a Tukey post hoc test.

Sodium azide sensitivity tests were conducted as described in [77]. At least 20 young adult animals of each condition were transferred to unseeded NGM plates containing 0.5 mM sodium azide and monitored for paralysis every 30 minutes. When a worm did not respond to a gentle prod with a platinum wire, it was marked as paralyzed. Missing animals, or animals that died because of vulva rupture, were censored from the analysis. Two populations were assayed per condition. Data were analyzed using a two-way ANOVA with a Dunnett post hoc test.

### Staining of cuticle components with DiI and WGA

The annuli of the cuticle were stained with DiI as described in [78]. Young adult animals were washed thrice in M9 buffer before staining with 30 μg/mL DiI dissolved in M9 for 12 hours. After staining, animals were washed once in M9 and imaged on a confocal microscope as described above to determine annuli width. The width of at least 10 annuli was determined per animal, and at least 7 animals per condition were imaged. The results were analyzed using a one-way ANOVA with a Tukey post hoc test. The cuticular surface was stained with rhodamine-conjugated WGA as described in [77,79]. Young adult animals were washed thrice in M9 buffer to remove residual bacteria and afterwards incubated in 200 μg/mL rhodamine-conjugated WGA dissolved in M9. After staining, animals were washed four times in M9 and imaged as described above. At least 12 animals were imaged per condition.

### TEM

Preparation of TEM samples was performed as described in [80]. Day 1 adult animals were rinsed off culture plates and fixed in cold glutaraldehyde (2%, pH 7.3) with 50 mM Na-cacodylate and 150 mM saccharose, followed by fixation in 2% (w/v) osmium tetroxide. Fixed worms were dehydrated in an acetone series and embedded in araldite. Semi-thin sections of 1 μm cut with a Reichert Ultracut E microtome (Ametek, Berwyn, PA) were stained with methylene blue and viewed in a DM300 light microscope (Leica, Germany) for orientation. Double-stained 70-nm thin sections were visualized using an EM900 transmission electron microscope (Zeiss, Germany).

### Mating contact assays

Mating contact assays were performed as described in [36]. Four day 1 adult hermaphrodite wild-type or mutant animals together with 8 day 1 adult wild-type males were picked onto a 55-mm NGM plate, seeded with a single 25-μL drop of OP50 *E*. *coli* grown in LB medium, creating a 10-mm circular spot of bacteria. After an adjustment period of at least 1 hour, the mating occupancy of each hermaphrodite was scored as either 1 (in contact with a male) or 0 (not in contact). Mating scores were summed up for each of the 4 hermaphrodites. This measurement was repeated every minute for 25 minutes, generating a total mating score indicative of the amount of time animals had spent in mating contact. At least three populations were assayed for each condition. Results were analyzed using a one-way ANOVA with a Dunnett post hoc test.

### Oxidative stress assays

Oxidative stress survival assays were performed essentially as described in [81]. Per condition, at least 3 populations consisting of approximately 20 day 1 adult animals were incubated in 500 μL M9 buffer solution supplemented with H_2_O_2_ (Sigma–Aldrich, St. Louis, MO) to a final concentration of 5 mM in a 24-well plate. Survival of each animal was assayed every hour by checking for movement or pharyngeal pumping. Missing animals, or animals that died because of vulva rupture, were censored in the analysis. Data were analyzed using a two-way ANOVA with a Dunnett post hoc test.

### Quantification of in vivo ROS permeation and redox state

Measurement of ROS permeation was performed using the Amplex Red hydrogen peroxide kit (Thermo Fisher Scientific, Waltham, MA) according to the manufacturer’s instructions and modified for use in *C*. *elegans* as described in [72]. A 50-μL worm pellet of day 1 adult animals was suspended and washed in the manufacturer’s reaction buffer and subsequently incubated while rotating in the dark in Amplex Red working solution for 1 hour. The absorbance intensity of the supernatant, as a measure of permeated ROS, was measured in a Greiner flat-bottom 96-well plate using a Tecan Infinite M200 plate reader with excitation and emission wavelengths set to 550 and 590 nm, respectively. At least 6 populations were measured per condition. Means were compared using a Student *t* test.

Measurement of in vivo redox state was performed as previously described [40,82,83]. *ceh-60* mutant animals were crossed with the JV10 RoGFP marker strain carrying the *rps-0p*::*roGFP2-Orp1* redox-sensitive *roGFP2* transgene under the ubiquitous ribosomal *rps-0* promoter. Day 1 adult wild-type and *ceh-60* mutant animals carrying the roGFP2 transgene were washed in physiological buffer and diluted to a concentration of 10 worms per μL. A total of 100 μL of worm suspension was added to each well of a flat-bottom 96-well plate (Sigma-Aldrich, St. Louis, MO) and fluorescence was recorded for 1 hour with excitation filters for oxidized (405 nm) and reduced (490 nm) roGFP2 and an emission wavelength at 535 nm using an Infinite M200 plate reader (Tecan, Switzerland). At each time point, the ratio of oxidized over reduced GFP was calculated as described in [84] and averaged over 1 hour. At least 12 populations of approximately 1,000 worms each were tested per condition. Means were compared using a Student *t* test. To test the sensitivity of the assay and the redox shift upon encounter of an exogenous stressor, H_2_O_2_ was added to a final concentration of 5 mM, and measurements of oxidized and reduced RoGFP intensity were taken for another 140 cycles.

## Supporting information

S1 Fig Lipids accumulate in the intestine of *ceh-60* adults but are less abundant in their embryos. (A) Quantification of Oil-red-O staining shows that lipids accumulate faster in the intestine of adult *ceh-60* (■) animals when compared with the wild type (●), a difference that becomes significant from day 3 of adulthood onwards. Staining intensity is relative to wild-type day 1, which is set at 100. Error bars: SEM, and ****p < 0.0001. *N* ≥ 5 for each time point. Underlying data are available in S1 Data. (B) Representative images of day 3 adults stained with Oil-red-O show that the intestinal region is more intensely stained in *ceh-60* animals. Yellow circles indicate regions used for intestinal fat quantification. Arrows indicate embryos inside the adult hermaphrodite. Scale bar, 200 μm.
(TIF)

S2 Fig *ceh-60* is expressed in AWC neurons, pharyngeal muscle, and intestine. (A) Expression of *ceh-60* (green, *ceh-60p*::*ceh-60*::*gfp*) and *odr-1* (red, *odr-1p*::*rfp* is AWC-specific [85]) overlaps, showing that *ceh-60* is expressed in the AWC neurons. (B,C) Bright-field and GFP images of intestinal (*) and pharyngeal (arrow) expression of *ceh-60* in strains carrying (B) a *ceh-60p*::*ceh-60*::*gfp* fosmid or (C) a *ceh-60p*::*ceh-60*::*SL2*::*gfp* construct. While pharyngeal expression is visible throughout life, its localization does not appear to be exclusively nuclear. Neuronal expression is always visible, marked with dotted circles. Scale bars, 20 μm. GFP, green fluorescent protein.
(TIF)

S3 Fig CEH-60 is not necessary for control of seam cell division. (A) Overlaid bright-field and fluorescence images of wild-type and *ceh-60* L4 animals carrying an integrated seam cell *gfp* marker (*SCMp*::*gfp*). Each animal has 16 seam cells visible on either side of the body. Scale bars, 100 μm. (B) Graph indicating the number of seam cells in wild-type, *ceh-60* mutant and *unc-62* RNAi-treated animals (positive control). There is no significant difference between *ceh-60* and wild-type animals, while *unc-62* RNAi-treated animals show modest seam cell hyperplasia. Error bars: SEM, and ***p < 0.001. *N* ≥ 6. Underlying data are available in S1 Data. *gfp*, green fluorescent protein; RNAi, RNA interference.
(TIF)

S4 Fig Knockdown of collagen genes does not cause cuticle hyperpermeability. Performing RNAi knockdown of *col-106* or *col-120* does not change the permeability of animals to acridine orange. Fluorescence intensity is relative to the wild type and is shown on a logarithmic scale. Error bars: SEM, and NS = not significant. *N* ≥ 36. Underlying data are available in S1 Data. *col*, collagen; RNAi, RNA interference.
(TIF)

S5 Fig Characterization of different cuticular components with DiI, WGA, and *col-19*::*gfp* reporter. (A) DiI staining of the annuli of the wild type, *ceh-60(lst466)*, and *ceh-60(ok1485)* shows that there is no difference in annuli morphology. Annuli width is relative to the average value for wild type, set as 100. Scale bar, 20 μm. Error bars: SEM, and NS = not significant. *N* ≥ 7. (B) Rhodamine-conjugated WGA stains *ceh-60* mutant animals but not wild types. Scale bar, 200 μm. Graph scale, logarithmic; error bars: SEM, and ****p < 0.0001. *N* ≥ 12. Underlying data for panel A and B are available in S1 Data. (C) Visualization of the cortical layer of the cuticle with *col-19*::*gfp* marker. Scale bar, 10 μm. DiI, 1,19-dioctadecyl-3,3,39,39-tetramethylindocarbocyanine perchlorate; WGA, wheat germ agglutinin.
(TIF)

S6 Fig Intestinal *unc-62* knockdown increases susceptibility to oxidative stress. Upon knockdown of *unc-62* (⬡) in the intestine-specific RNAi strain MGH167, animals become more susceptible to oxidative stress than empty vector–treated animals (●), although the effect is slightly less pronounced than in *ceh-60(lst466)* mutants (■). Error bars: SEM. ***p < 0.001. *N* ≥ 4. Underlying data are available in S1 Data. RNAi, RNA interference.
(TIF)

S7 Fig CEH-60 affects sensitivity to sodium azide. Sodium azide sensitivity as measured by fraction of worms moving during incubation in 0.5 mM NaN_3_ is lower in *ceh-60* mutant animals than in control animals. This defect is rescued by intestinal expression of *ceh-60* (*elt-2p*::*ceh-60*), but not by expression of *ceh-60* with a truncated PBC-interaction domain (*ceh-60p*::*ceh-60(ΔPBC)*). Error bars indicate SEM. **p < 0.01, ****p < 0.0001. *N* = 2. Underlying data are available in S1 Data. PBC, pre–B cell leukemia.
(TIF)

S8 Fig *ceh-60* mRNA levels oscillate with molting. mRNA abundance of *ceh-60* (■) cycles during development, apparently peaking each time at the end of a molt, as deduced from the *lin-42* expression profile. Molts are recognized as local minima in *lin-42* expression (gray line) [86]. Adopted under creative commons license 4.0 from [3], where it is also shown that *ceh-60* expression dramatically increases during the final larval molt (as indicated by the dashed arrow). Underlying data are available in S1 Data.
(TIF)

S9 Fig Ratio of oxidized over reduced RoGFP2 before and after addition of exogenous stress. Wild-type (─) and *ceh-60* (gray line) animals show no difference in redox state when observed under unstressed conditions, but when an exogenous stressor in the form of 5 mM H_2_O_2_ is added after 20 cycles (indicated by arrow), their ratio of oxidized/reduced RoGFP2 increases more than in wild-type animals. *N* ≥ 8. Underlying data are available in S1 Data. RoGFP2, reduction-oxidation sensitive green fluorescent protein.
(TIF)

S1 Data Data underlying Figs 2–7, S1 Fig and S3–S9 Figs. (XLSX)

## Abbreviations

ACDH-1: acyl-coenzyme A dehydrogenase 1
AWC: amphid wing “C”
BiFC: bimolecular fluorescence complementation
bus: bacterially un-swollen
CEH-60: *C*. *elegans* homeobox 60
CID: collision-induced dissociation
CLEC-63: C-type lectin 63
CoA: coenzyme A
COL: collagen
DiI: 1,19-dioctadecyl-3,3,39,39-tetramethylindocarbocyanine perchlorate
ELT-2: erythroid-like transcription factor family 2
EXD: extradenticle
GFP: green fluorescent protein
GST-7: glutathione S-transferase 7
HACH-1: hydroxyacyl-coenzyme A hydrolase 1
HCD: high-energy collision-induced dissociation
HTH: homothorax
IPTG: isopropyl-β-D-thiogalactoside
IVD-1: isovaleryl- coenzyme A dehydrogenase 1
LIN-29: abnormal cell lineage 29
MAB-3: male abnormal 3
MEIS: myeloid ecotropic viral integration site
miRNA: micro ribonucleic acid
NGM: nematode growth medium
PBC: pre–B cell leukemia
PBX: pre–B cell leukemia homeobox
PGP-1: P-glycoProtein related 1
PQM-1: paraquat (methylviologen) responsive 1
PREP: PBX (pre–B cell leukemia transcription factor) regulating protein
RNAi: ribonucleic acid interference
RNAseq: ribonucleic acid sequencing
RoGFP2: reduction-oxidation sensitive green fluorescent protein 2
ROS: reactive oxygen species
SGK-1: serum- and glucocorticoid- inducible kinase homolog 1
TALE: three amino acid loop extension
TEM: transmission electron microscopy
TORC-2: target of rapamycin complex 2
UNC-62: uncoordinated 62
VIT: vitellogenin
WGA: wheat germ agglutinin
