# Supplementary figures and images for: CEH-60/PBX regulates vitellogenesis and cuticle permeability through intestinal interaction with UNC-62/MEIS in Caenorhabditis elegans
Source: PLoS Biol. 2019 Nov 1;17(11):e3000499. doi: 10.1371/journal.pbio.3000499 (PMC6824563; doi:10.1371/journal.pbio.3000499)

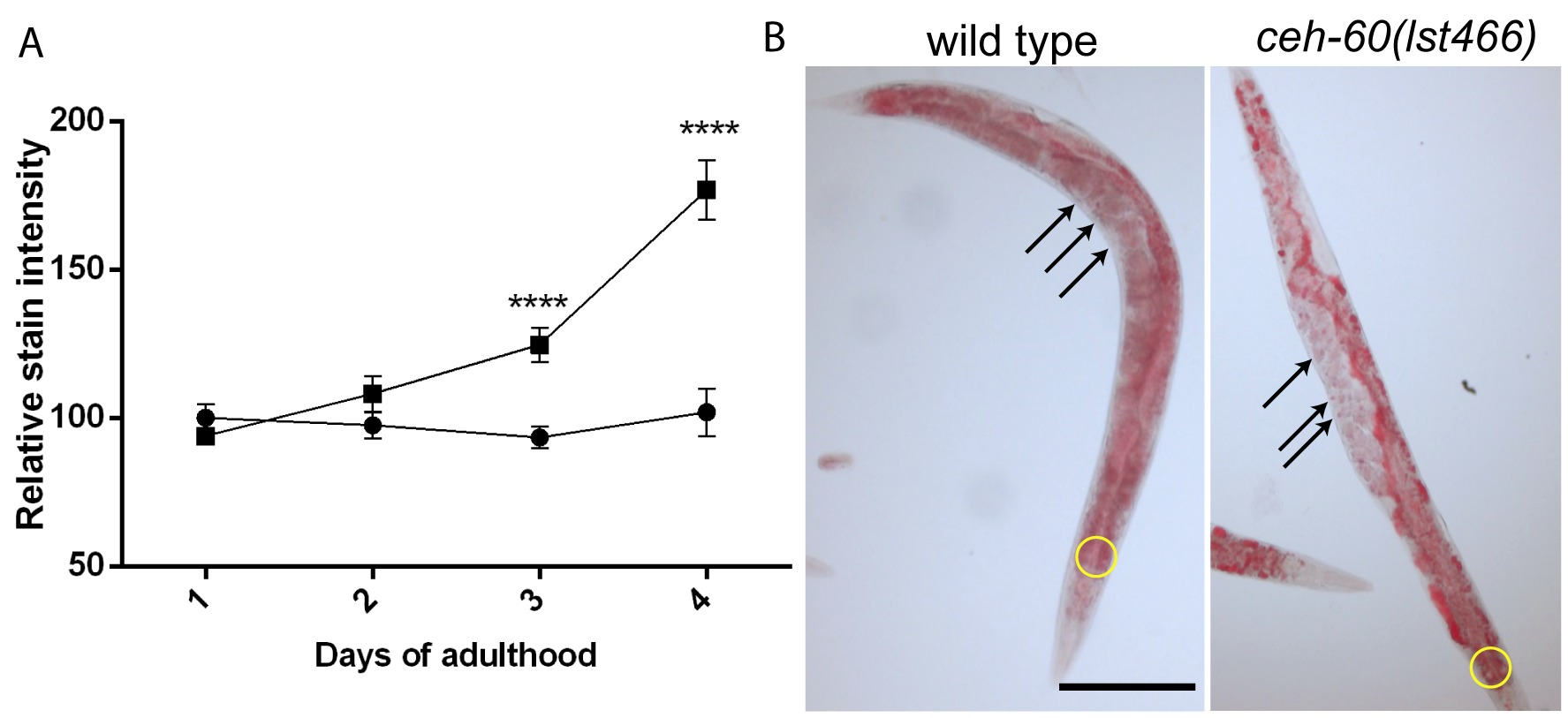

Supplement: S1 Fig — (A) Quantification of Oil-red-O staining shows that lipids accumulate faster in the intestine of adult ceh-60 (■) animals when compared with the wild type (●), a difference that becomes significant from day 3 of adulthood onwards. Staining intensity is relative to wild-type day 1, which is set at 100. Error bars: SEM, and ****p < 0.0001. N ≥ 5 for each time point. Underlying data are available in S1 Data. (B) Representative images of day 3 adults stained with Oil-red-O show that the intestinal region is more intensely stained in ceh-60 animals. Yellow circles indicate regions used for intestinal fat quantification. Arrows indicate embryos inside the adult hermaphrodite. Scale bar, 200 μm. (TIF) [file pbio.3000499.s001.tif]

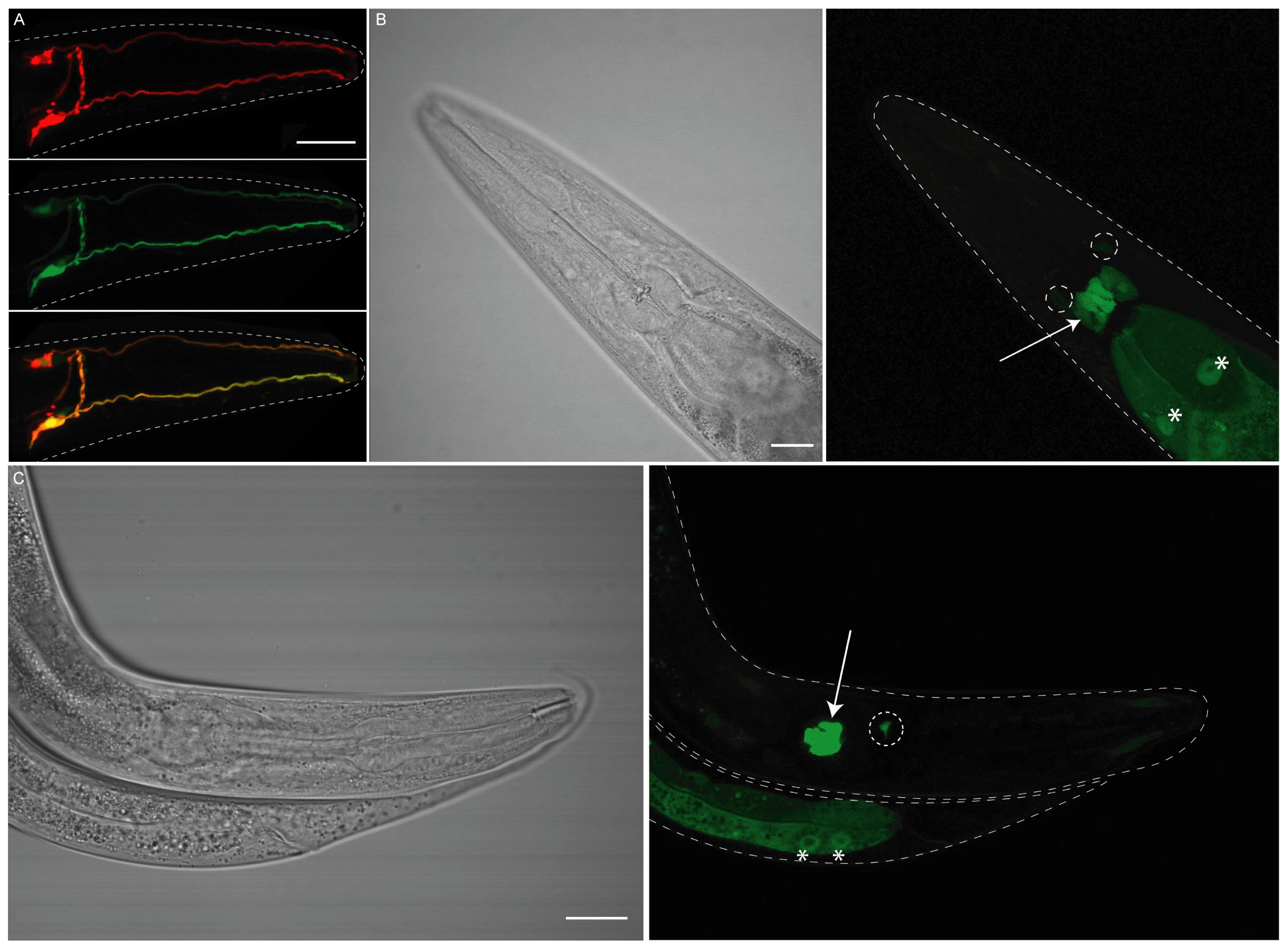

Supplement: S2 Fig — (A) Expression of ceh-60 (green, ceh-60p::ceh-60::gfp) and odr-1 (red, odr-1p::rfp is AWC-specific [85]) overlaps, showing that ceh-60 is expressed in the AWC neurons. (B,C) Bright-field and GFP images of intestinal (*) and pharyngeal (arrow) expression of ceh-60 in strains carrying (B) a ceh-60p::ceh-60::gfp fosmid or (C) a ceh-60p::ceh-60::SL2::gfp construct. While pharyngeal expression is visible throughout life, its localization does not appear to be exclusively nuclear. Neuronal expression is always visible, marked with dotted circles. Scale bars, 20 μm. GFP, green fluorescent protein. (TIF) [file pbio.3000499.s002.tif]

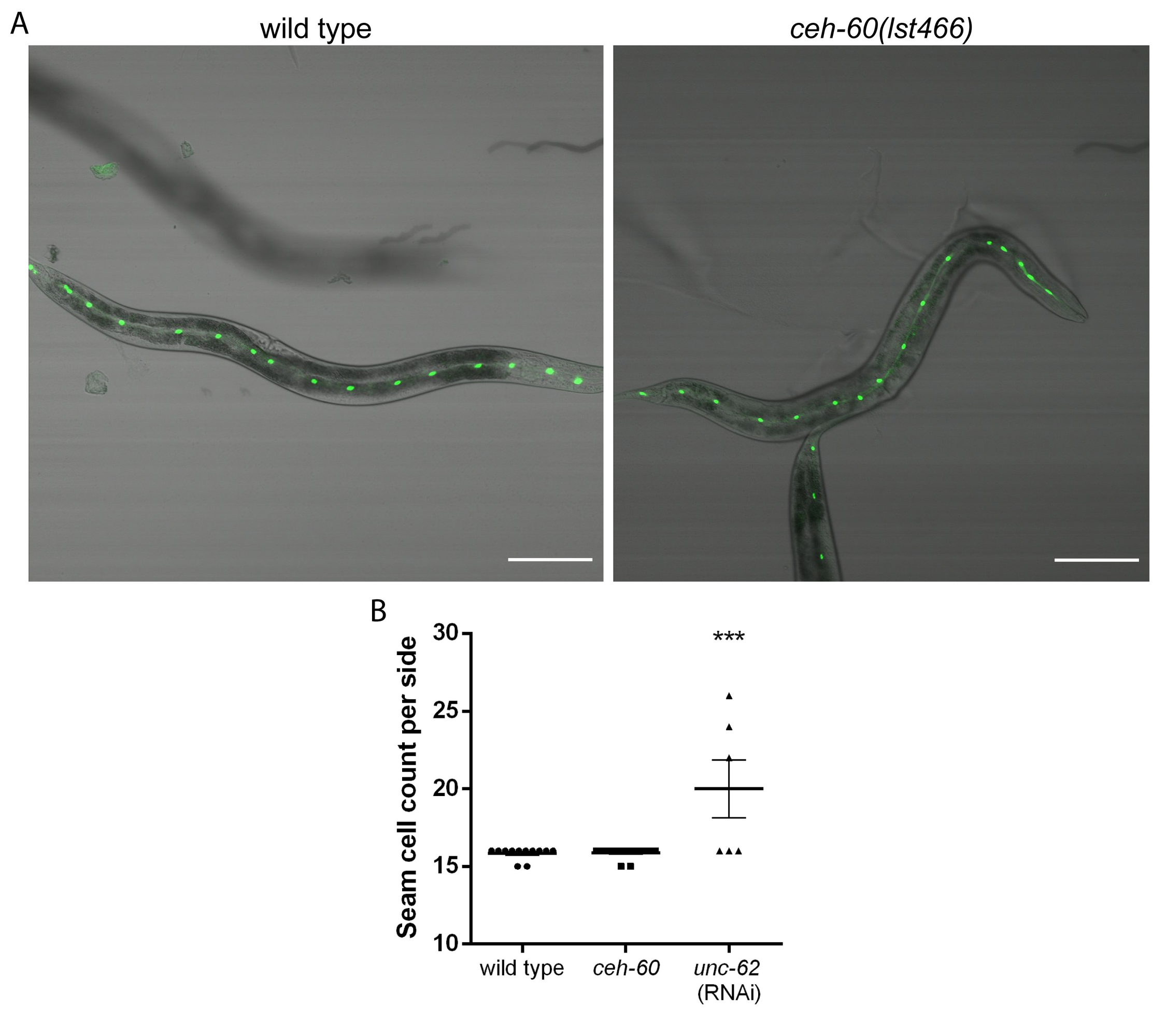

Supplement: S3 Fig — (A) Overlaid bright-field and fluorescence images of wild-type and ceh-60 L4 animals carrying an integrated seam cell gfp marker (SCMp::gfp). Each animal has 16 seam cells visible on either side of the body. Scale bars, 100 μm. (B) Graph indicating the number of seam cells in wild-type, ceh-60 mutant and unc-62 RNAi-treated animals (positive control). There is no significant difference between ceh-60 and wild-type animals, while unc-62 RNAi-treated animals show modest seam cell hyperplasia. Error bars: SEM, and ***p < 0.001. N ≥ 6. Underlying data are available in S1 Data. gfp, green fluorescent protein; RNAi, RNA interference. (TIF) [file pbio.3000499.s003.tif]

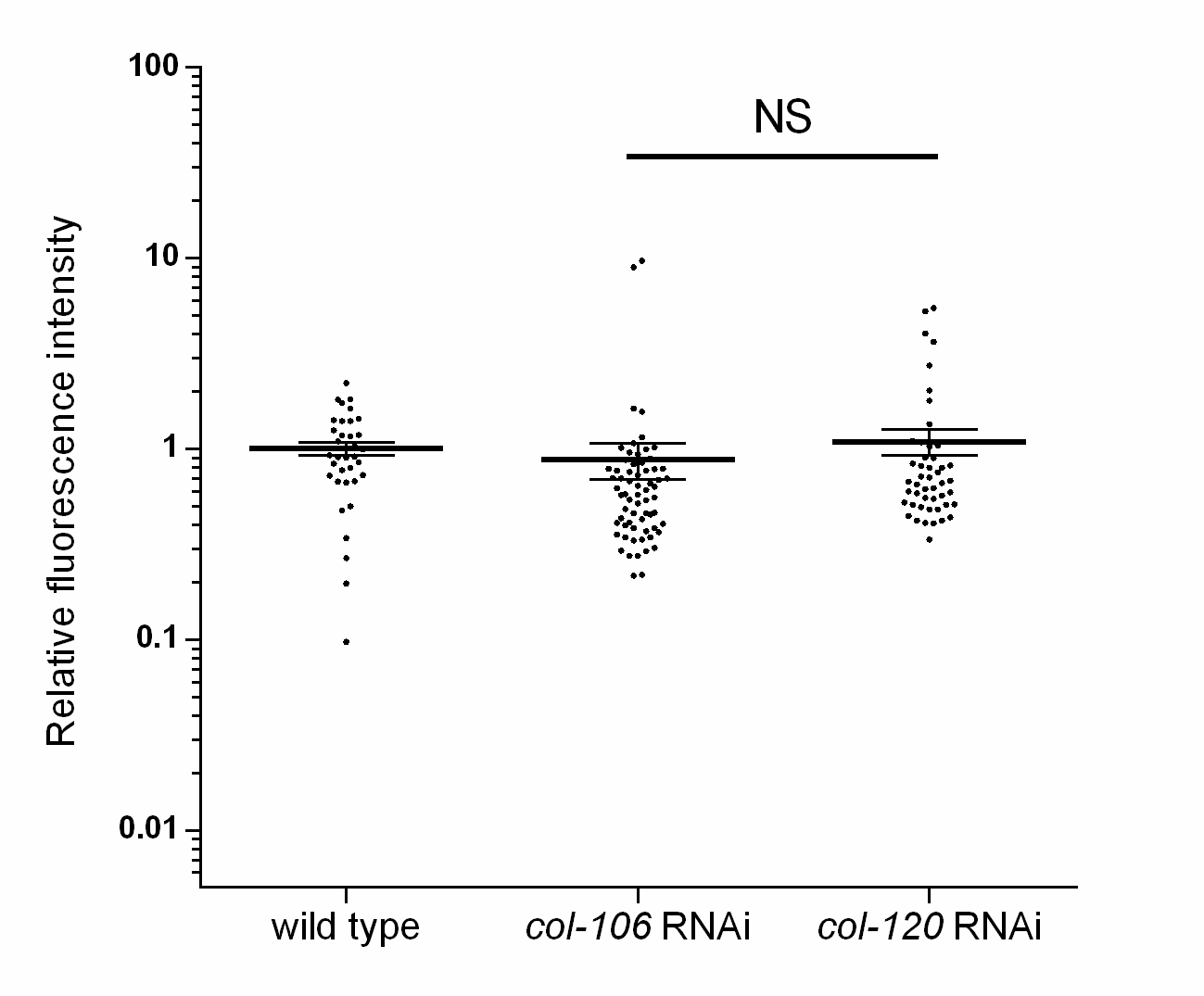

Supplement: S4 Fig — Performing RNAi knockdown of col-106 or col-120 does not change the permeability of animals to acridine orange. Fluorescence intensity is relative to the wild type and is shown on a logarithmic scale. Error bars: SEM, and NS = not significant. N ≥ 36. Underlying data are available in S1 Data. col, collagen; RNAi, RNA interference. (TIF) [file pbio.3000499.s004.tif]

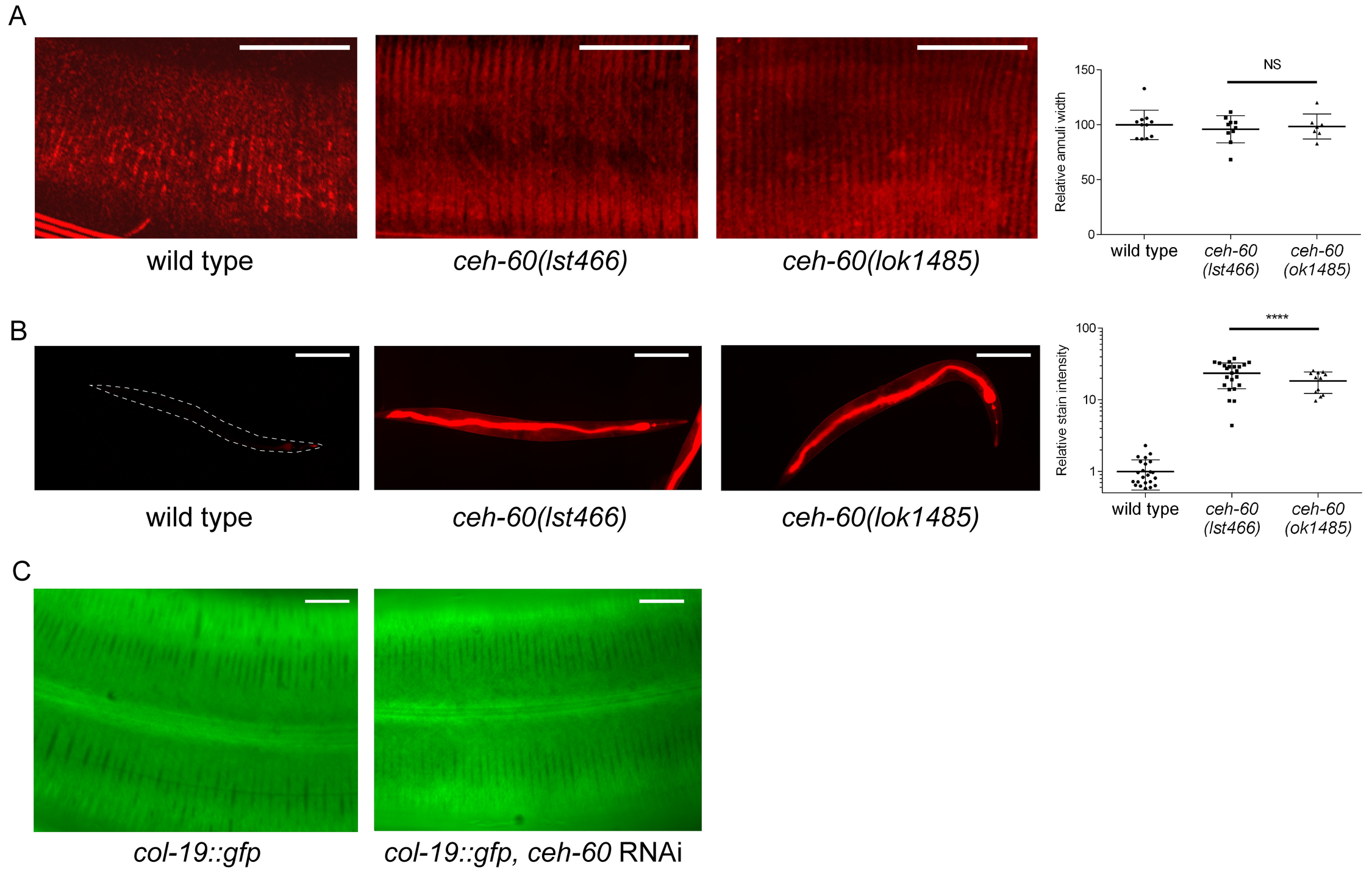

Supplement: S5 Fig — (A) DiI staining of the annuli of the wild type, ceh-60(lst466), and ceh-60(ok1485) shows that there is no difference in annuli morphology. Annuli width is relative to the average value for wild type, set as 100. Scale bar, 20 μm. Error bars: SEM, and NS = not significant. N ≥ 7. (B) Rhodamine-conjugated WGA stains ceh-60 mutant animals but not wild types. Scale bar, 200 μm. Graph scale, logarithmic; error bars: SEM, and ****p < 0.0001. N ≥ 12. Underlying data for panel A and B are available in S1 Data. (C) Visualization of the cortical layer of the cuticle with col-19::gfp marker. Scale bar, 10 μm. DiI, 1,19-dioctadecyl-3,3,39,39-tetramethylindocarbocyanine perchlorate; WGA, wheat germ agglutinin. (TIF) [file pbio.3000499.s005.tif]

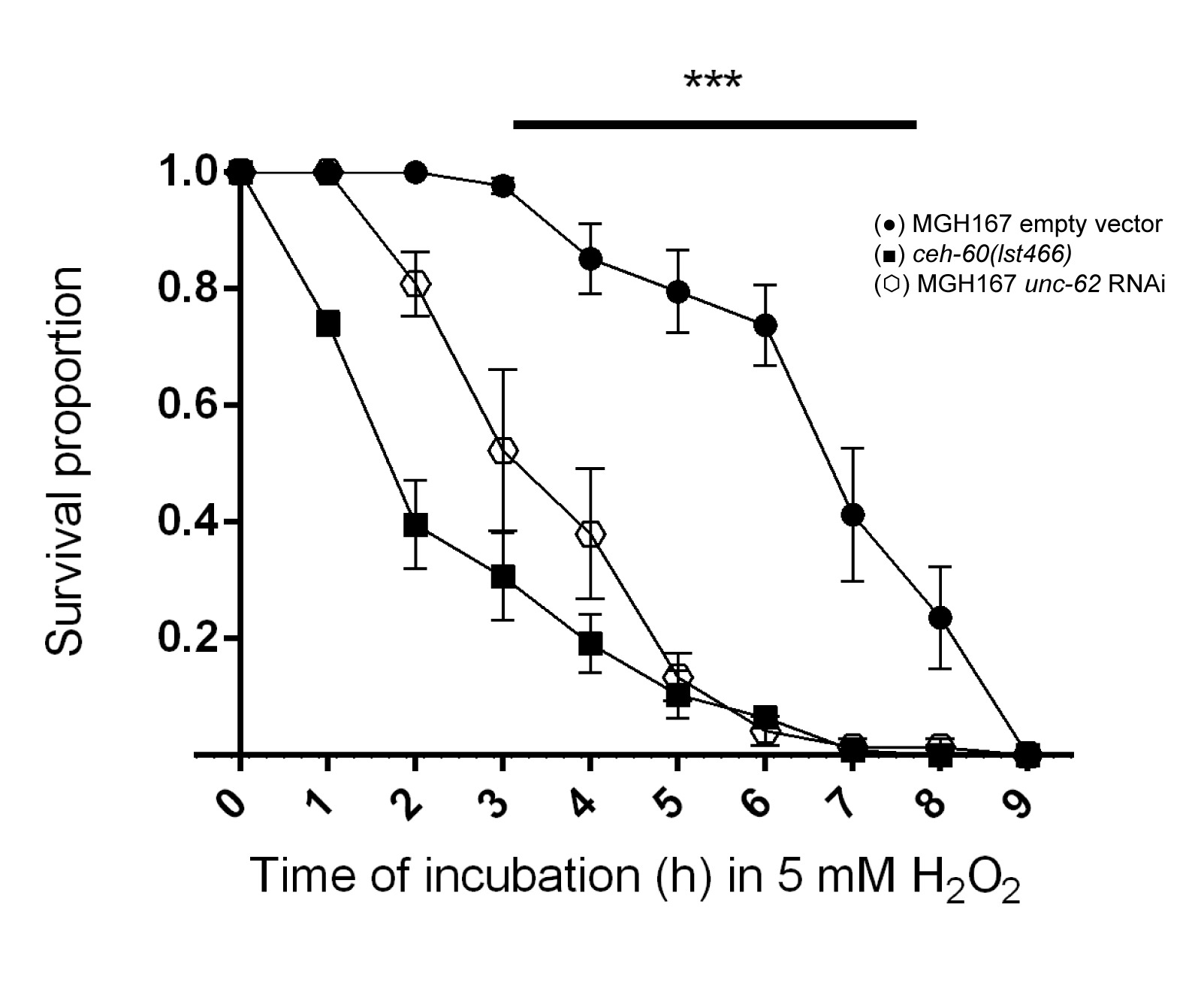

Supplement: S6 Fig — Upon knockdown of unc-62 (⬡) in the intestine-specific RNAi strain MGH167, animals become more susceptible to oxidative stress than empty vector–treated animals (●), although the effect is slightly less pronounced than in ceh-60(lst466) mutants (■). Error bars: SEM. ***p < 0.001. N ≥ 4. Underlying data are available in S1 Data. RNAi, RNA interference. (TIF) [file pbio.3000499.s006.tif]

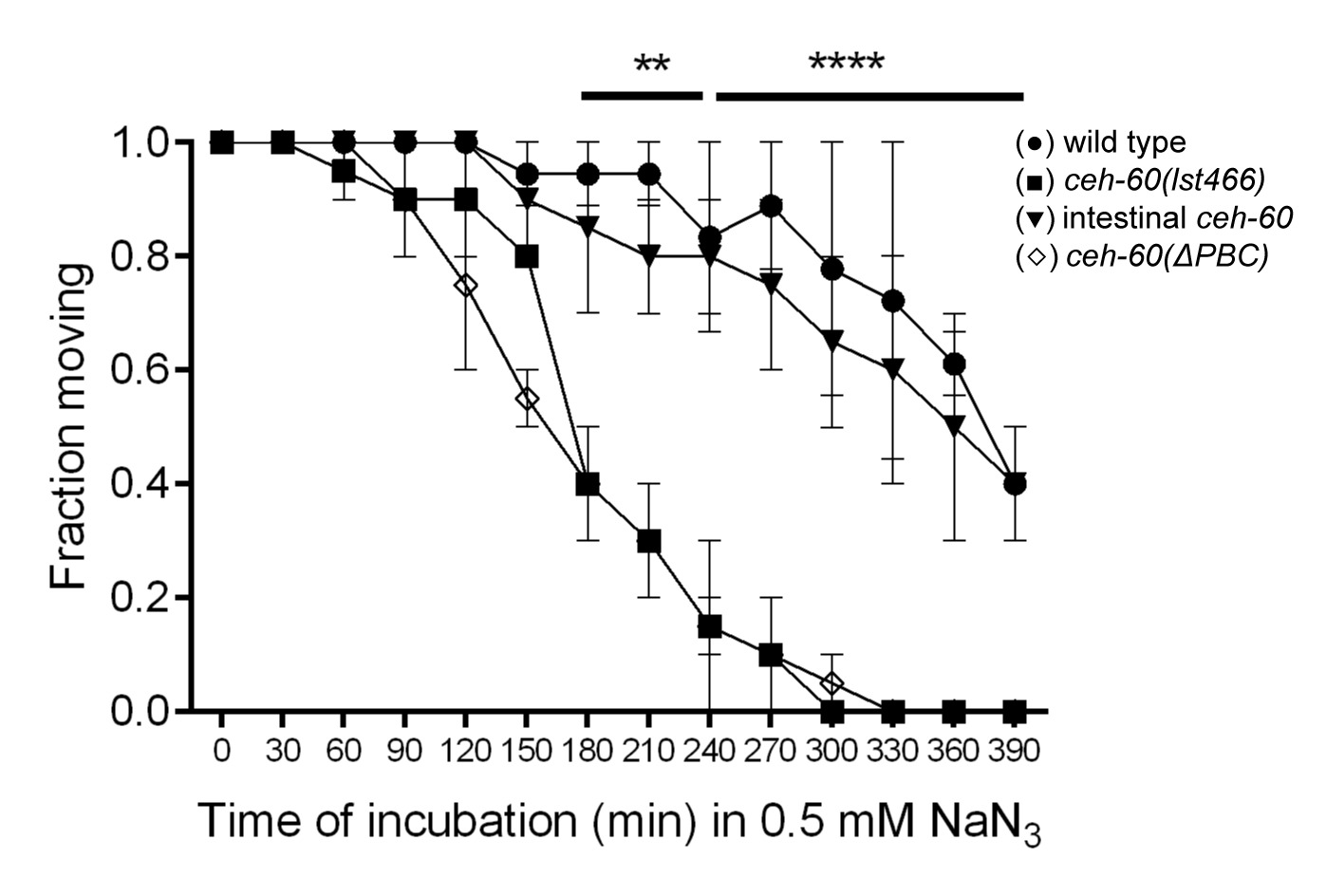

Supplement: S7 Fig — Sodium azide sensitivity as measured by fraction of worms moving during incubation in 0.5 mM NaN3 is lower in ceh-60 mutant animals than in control animals. This defect is rescued by intestinal expression of ceh-60 (elt-2p::ceh-60), but not by expression of ceh-60 with a truncated PBC-interaction domain (ceh-60p::ceh-60(ΔPBC)). Error bars indicate SEM. **p < 0.01, ****p < 0.0001. N = 2. Underlying data are available in S1 Data. PBC, pre–B cell leukemia. (TIF) [file pbio.3000499.s007.tif]

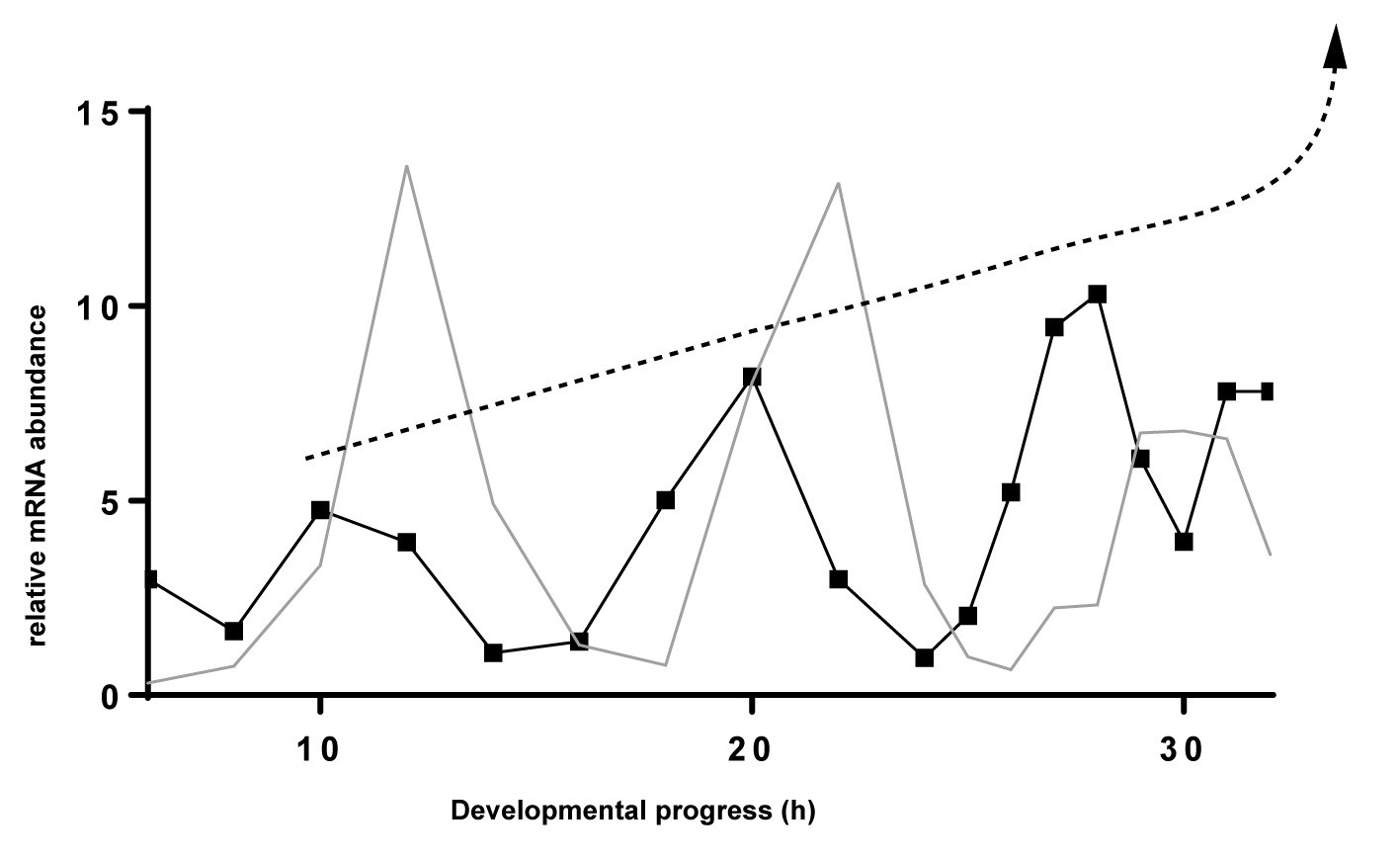

Supplement: S8 Fig — mRNA abundance of ceh-60 (■) cycles during development, apparently peaking each time at the end of a molt, as deduced from the lin-42 expression profile. Molts are recognized as local minima in lin-42 expression (gray line) [86]. Adopted under creative commons license 4.0 from [3], where it is also shown that ceh-60 expression dramatically increases during the final larval molt (as indicated by the dashed arrow). Underlying data are available in S1 Data. (TIF) [file pbio.3000499.s008.tif]

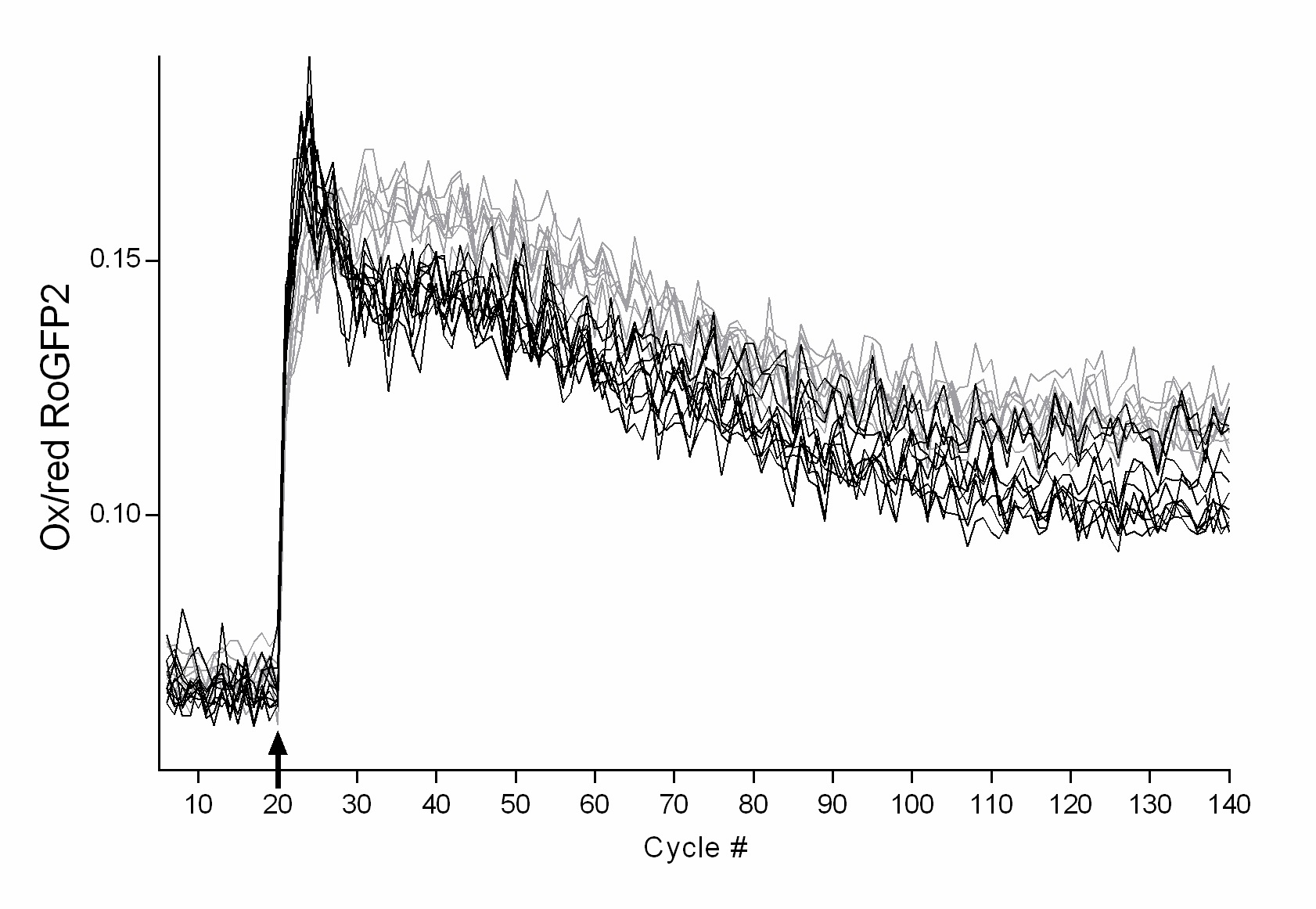

Supplement: S9 Fig — Wild-type (─) and ceh-60 (gray line) animals show no difference in redox state when observed under unstressed conditions, but when an exogenous stressor in the form of 5 mM H2O2 is added after 20 cycles (indicated by arrow), their ratio of oxidized/reduced RoGFP2 increases more than in wild-type animals. N ≥ 8. Underlying data are available in S1 Data. RoGFP2, reduction-oxidation sensitive green fluorescent protein. (TIF) [file pbio.3000499.s009.tif]
